# Mechanistic insights into SIRT7 and EZH2 regulation of cisplatin resistance in bladder cancer cells

**DOI:** 10.1038/s41419-024-07321-1

**Published:** 2024-12-24

**Authors:** Yudong Cao, Shuo Wang, Jinchao Ma, Mengping Long, Xiuli Ma, Xiao Yang, Yongpeng Ji, Xingxing Tang, Jia Liu, Chen Lin, Yong Yang, Peng Du

**Affiliations:** 1 Key laboratory of Carcinogenesis and Translational Research (Ministry of Education/Beijing), Department of Urology, Peking University Cancer Hospital & Institute, Beijing, 100142 China; 2 Key laboratory of Carcinogenesis and Translational Research (Ministry of Education/Beijing), Department of Pathology, Peking University Cancer Hospital & Institute, Beijing, 100142 China

**Keywords:** Epigenetics, Cancer therapeutic resistance, Bladder cancer

## Abstract

Cisplatin (CDDP) resistance has been established to significantly impact Bladder Cancer (BCa) therapy. On the other hand, the crucial regulatory involvement of SIRT7 and EZH2 in bladder cancer development is well known. Herein, the collaborative regulatory roles and underlying mechanisms of SIRT7 and EZH2 in CDDP resistance in bladder cancer were explored. Immunohistochemistry (IHC) and Western Blot (WB) analyses were used to assess the expression levels of SIRT7/EZH2 and RND3 in bladder cancer tissues, normal ureteral epithelial cells, and bladder cancer cell lines. Furthermore, the impact of various treatments on of UMUC3 cell proliferation and CDDP sensitivity was assessed using CCK-8 assays, plate cloning assays, and flow cytometry analysis. Additionally, the levels of H3K18ac and H3K27me^3^ at the promoter region of the RND3 gene, the binding abilities of SIRT7 and EZH2, and the succinylation level of the EZH2 protein were examined using ChIP-qPCR assays, CO-IP assays, and IP assays, respectively. Moreover, in vivo experiments were conducted using a bladder cancer mouse model created by subcutaneously injecting UMUC3 cells into Balb/c nude mice. According to the results, SIRT7 correlated with the sensitivity of bladder cancer cells to both the platinum-based chemotherapy and CDDP. Specifically, SIRT7 could bind to the RND3 promoter, downregulating H3K18ac and RND3, ultimately leading to an increased CDDP sensitivity in UMUC3 cells. Furthermore, EZH2 siRNA could decrease H3K27me^3^ levels in the RND3 promoter, upregulating RND3. Overall, in the promoter region of the RND3 gene, SIRT7 upregulated H3K27me^3^ and EZH2 downregulated H3K18ac, leading to a decline in RND3 expression and CDDP sensitivity in bladder cancer cells. Additionally, SIRT7 reduced the succinylation of the EZH2 protein resulting in an EZH2-mediated RND3 downregulation. Therefore, targeting SIRT7 and EZH2 could be a viable approach to enhancing CDDP efficacy in bladder cancer treatment.

## Introduction

Bladder Cancer (BCa), the second most prevalent malignancy in the human urogenital system, has the highest mortality rate, attributable to its high recurrence rate and extensive resistance to anticancer medications [1]. Presently, platinum-based chemotherapy is the first-line treatment for muscle-invasive and metastatic bladder cancer. Some of the platinum-based medications include Cisplatin (CDPP), carboplatin, and oxaliplatin. Notably, although 60–70% of CDPP-treated BCa patients exhibited a preliminary response to the drug, the Progression-Free Survival (PFS) and Overall Survival (OS) of most CDPP-treated BCa patients are often not satisfactory due to reduced responsiveness in the short term. This shortcoming remains a major challenge in BCa treatment of this disease [2]. Therefore, identifying the key targets that mediate CDDP sensitivity is crucial in BCa treatment, as well as in developing new therapeutic strategies for refractory bladder cancer [3].

Sirtuins (SIRTs), highly conserved enzymes known to target both histone and nonhistone proteins, are a family of NAD^+^-dependent deacetylases with seven members (SIRT1-SIRT7) [4]. Notably, mammalian SIRTs have been associated with multiple biological processes, including cellular stress resistance, tumorigenesis, and energy metabolism [5]. For instance, recent research has shown a correlation between altered SIRT7 expression and various human cancers, indicating its significant involvement in cellular processes that could contribute to oncogenic transformation and tumor biology [6, 7]. Furthermore, bladder urothelial carcinoma patients exhibited a significantly higher SIRT7 expression than normal mucosae cohorts [8]. Additionally, besides increased BCa cell proliferation, SIRT7 has also been associated with Epithelial-Mesenchymal Transition (EMT) [8, 9]. Although the regulatory role of SIRT7 in CDDP sensitivity in cancer cells is yet to be explored, SIRT7 has been established to be involved in CDDP-mediated kidney injury [10, 11]. According to research, SIRT7 functions as an NAD (^+^)-dependent deacetylase of histone H3 acetylated lysine 18 (H3K18ac), causing the stabilization of the oncogenic phenotype in cancer cells and facilitating transcriptional repression [12]. Research has also revealed that CDDP can inhibit SIRT7-mediated H3K18 deacetylation in renal cell HEK293T cells [13]. However, whether SIRT7 affects CDDP sensitivity in BCa cells remains unclear.

The histone methyltransferase enhancer of zeste homolog 2 (EZH2) serves as the enzymatic catalytic subunit of the Polycomb-Repressive Complex 2 (PRC2), modulating gene expression via trimethylation of lysine 27 on histone 3 (H3K27). According to research, EZH2 is involved in global transcriptional suppression and is overexpressed in multiple tumor entities, including ovarian [14], breast [15], bladder [16], and renal [16] malignancies; hence, it is classified as an oncogene. Moreover, studies have indicated that EZH2 suppresses the expression of genes such as CBX7 [17] and miR-194-5p [18], diminishing the sensitivity of BCa cells to CDDP. Notably, increasing evidence suggests that epigenetic regulatory enzymes can collaboratively modulate gene transcription activity via epigenetic modifications. For example, in Nonalcoholic Fatty Liver Disease (NAFLD)-related Hepatocellular Carcinoma (HCC), HDAC8 and EZH2 regulated H4ac content and H3K27me^3^ levels of the AXIN2 gene, respectively. Furthermore, they jointly silenced AXIN2 expression, ultimately promote the activation of the proto-oncogenic pathway, β-catenin pathway [19]. Additionally, in HCC, SIRT7 and EZH2 affected H3K18ac levels and H3K27me^3^ content of the genome, respectively. They also jointly induced gene expression silencing, exerting a collaborative regulatory effect [20]. Nonetheless, it remains unclear whether EZH2 may cooperate with SIRT7 to silence the transcription expression of tumor suppressor genes, as well as regulate BCa progression and CDDP sensitivity.

Herein, we found that SIRT7 and EZH2 can collaborate to regulate BCa cell growth and CDDP resistance. Mechanistically, RNA-sequencing and the qRT-PCR assay revealed that RND3 is the target of SIRT7. Furthermore, both SIRT7 and EZH2 could be recruited to the RND3 gene promoter region, respectively altering its H3K18ac and H3K27me^3^ content, thus leading to RND3 expression inhibition. Additionally, SIRT7 could reduce the succinylation level of EZH2 via binding interaction, lead to significant EZH2 upregulation at the RND3 gene promoter. Overall, targeting SIRT7 and EZH2 expression could lead to RND3 upregulation, enhancing BCa cell CDDP sensitivity.

## Methods

### Clinical sample acquisition

This study enrolled patients diagnosed with muscle-invasive bladder cancer and treated at Peking University Cancer Hospital Urology Surgery between April 2014 and April 2022, with postoperative pathological stage pT2-4N0-2M0, who underwent CDDP or carboplatin-based chemotherapy following radical cystectomy. Those included in the final analysis had complete clinical and pathological data (including pathological type, vascular invasion, etc.). Patients with other malignant tumors or those with severe heart disease, lung disease, and other conditions that threatened life in the short term were excluded. The patients were followed up postoperatively, receiving a review every three months for the first two years post-surgery, every six months for the third to fifth years, and every year after five years. During the review, abdominal and pelvic cavity enhanced CT or urological ultrasound, chest CT, urine routine, and bone scan or head CT were performed where necessary. In cases of suspected local recurrence or distant metastasis, further comprehensive PET/CT examination or puncture pathological biopsy were conducted verify the results. For patients who did not undergo follow-up visits at our hospital, their conditions were determined through telephone calls, which included their survival status and recurrence status. Clinical data were obtained from 126 bladder cancer patients, including disease-free survival (DFS), referring to the time of tumor recurrence post-operation, and overall survive (OS), as well as surgically removed bladder cancer tissues.

### Immunohistochemistry

Paraffin sections were treated were treated with a repair solution for antigen retrieval and then blocked with 3% hydrogen peroxide (Aladdin, H112515, Shanghai, CN) to inhibit endogenous peroxidase activity. This was followed by incubated with antibodies (SIRT7, Servicebio, GB11355-100, 1:200, Wuhan, CN; EZH2, Servicebio, GB12272-100, 1:200; RND3, Epigentek, A-6168-100, 1:200, Wuhan, CN) overnight. After washing with PBS, the secondary antibody (Goat Anti-Rabbit IgG (H + L) HRP, Affinity, S0001, 1: 200) was applied and left to incubate for 30 min. The DAB color development solution (Biosharp, BL732A, Hefei, CN) was added drop by drop, followed by counterstaining with Harris hematoxylin (Biosharp, BL702B). After the application of neutral adhesive, the samples were examined using a microscope (Olympus, BX53, Tokyo, Japan).

### Cell culture

The cell lines SV-HUC-1 (Procell, CL-0222, Wuhan, CN), T24 (Procell, CL-0227), UMUC3 (Procell, CL-0227), J82 (Procell, CL-0125), MGHU3 (Shjning, JN-CC5243, Shanghai, CN), TCCSUP (Procell, CL-0628), SW780 (Procell, CL-0449), and RT112 (Procell, CL-0682) were cultured under specific culture medium with 10% FBS (Procell, 164210-50) and 1% P/S (Procell, PB180120). The Ham’s F-12K (Procell, PM150910) was used to culture SV-HUC-1, McCoy’s 5 A (Procell, PM150710) for T24, whereas the MEM (containing NEAA, Procell, PM150410) was used for UMUC3, J82, MGHU3, and TCCSUP culture. The SW780 and RT112 cells were cultured in Leibovitz’s L-15 (Procell, PM151010) and RPMI-1640 (Procell, PM150110) media, respectively. The cells were cultured in a 37 °C, 5% CO_2_ incubator, with passages performed at a 1:3 ratio.

### Cell transfection

In our study, the Lipofectamine^TM^ 2000 was used to transfect the cells with small interfering RNA (siRNA) and overexpression plasmids into bladder cancer cells. Following trypsin digestion, 2 × 10^5^ cells were seeded into a 6-well plate for overnight incubation. The 20 μmol siRNA solution or 2 μg plasmids was thinned in 100 μL of serum-free opti-MEM and left to sit for 10 min and then added in order to the 6-well plate, mixed gently, and placed back in the incubator for further growth. At 48 h post-transfection, protein samples were extracted to verify the efficiency of siRNA and plasmids. Overexpression plasmids were extracted from Vectorbuilder (SIRT7, VB900090-9921jvu; EZH2, VB900001-6482umr; RND3, VB900000-4931mfx). The sequence of the siRNA (Genepharma, Shanghai, CN) is as follows: NC siRNA (Forward: UCGUAAGUAAGCGCAACCC, Reverse: GGGUUGCGCUUACUUACGA), SIRT7 siRNA1 (Forward: GCCUGAAGGUUCUAAAGAAGU, Reverse: UUCUUUAGAACCUUCAGGCUG), SIRT7 siRNA2 (Forward: CCUGCGUGCUGGUGAAGAAGG, Reverse: UUCUUCACCAGCACGCAGGGG), SIRT7 siRNA3 (Forward: CGGGAACAUGUACAUUGAAGU, Reverse: UUCAAUGUACAUGUUCCCGUG), RND3 siRNA1 (Forward: GACAGAUGUUAGUACAUUAGU, Reverse: UAAUGUACUAACAUCUGUCCG), RND3 siRNA2 (Forward: CGGACUUACGAAAGGACAAAG, Reverse: UUGUCCUUUCGUAAGUCCGUA), RND3 siRNA3 (Forward: AGAUGUUAGUACAUUAGUAGA, Reverse: UACUAAUGUACUAACAUCUGU), EZH2 siRNA1 (Forward: GGAUGGUACUUUCAUUGAAGA, Reverse: UUCAAUGAAAGUACCAUCCUG), EZH2 siRNA2 (Forward: GCAAAGUACUGUAAGAAUAAU, Reverse: UAUUCUUACAGUACUUUGCAA), EZH2 siRNA3 (Forward: CCAUGUUUACAACUAUCAACC, Reverse: UUGAUAGUUGUAAACAUGGUU).

### Cell Counting Kit-8 (CCK-8)

The bladder cancer cells were digested with trypsin, and then 100 μL of a single-cell suspension at a concentration of 5 × 10^4^/mL was added to each well of a 96-well plate. After cell attachment, CDDP (0, 0.5, 1, 2, 4, 8 and 16 μM, Sigma, 232120, Shanghai, CN) was added, followed by SIRT7 siRNA, RND3 siRNA, EZH2 siRNA, or siRNA NC transfection for 72 h. The culture medium was collected from the specified wells and washed with PBS. Next, 10 μL of CCK-8 (MCE, HY-K0301, Shanghai, CN) solution was added per well, and then incubated with the culture plate in an incubator for 2 h. The cell culture of medium was measures using a microplate reader (Thermo Fisher, Multiskan SkyHigh, Massachusetts, USA) to obtain the absorbance of each well at 450 nm.

### Clone formation

About 500 cells were evenly added into each well of a 6-well plate. Next, the cells were incubated at a 37 °C, 5% CO_2_ chamber for 2 weeks and treated with 4% paraformaldehyde for 15 min. This was followed by removal of the fixative and staining with 1 mL of Wright-Gimsa compound staining solution (Solarbio, G1020, Beijing, CN). Following a 20 min incubation period, the cells were rinsed to remove the staining solution and then air-dried at room temperature. Photos were taken and clone counts were subjected to further analysis.

### Cell apoptosis

After cell treatment, the cells were collected, rinsed twice with PBS, and centrifuged at 1000 rpm for 5 min. The AnnexinV-FITC/PI cell apoptosis detection kit (Nanjing KGA Biotech, KGA108, Nanjin, CN) was used to evaluate apoptosis of cells. Briefly, 500 μL Binding Buffer was added to the resuspended cells, and incubated with 5 μL AnnexinV-FITC. It was mixed and incubated with 5 μL PI to each well. Next, it was allowed to reach at room temperature in the dark for 10 min. The cell apoptosis was measured with a flow cytometer (CytoFLEX, BECKMAN, USA).

### Library construction and sequencing after RNA extraction

The RNA was extracted using TRIzol (Thermo Fisher). The levels of RNA, its quality, and condition were determined with ND-1000 (NanoDrop, MA, USA) and Bioanalyzer 2100(Agilent, CA, USA), to ensure that the concentrations were above 50 ng/μL, RIN values were higher than 7.0, and the total RNA exceeded 1 μg. PolyA+ mRNA was enriched using oligo (dT) beads through dual purification steps. The RNA fragments were generated using the magnesium fragmentation kit for further cDNA synthesis with the Invitrogen’s SuperScript II Reverse Transcriptase. *E. coli* DNA polymerase I and RNase H were used to synthesize double-stranded cDNA, and dUTP was added which was later removed. Adenine tailing and adapter ligation was then performed, followed by fragment selection and purification. 300 bp±50 bp strand-specific library was developed through the UDG enzyme digestion and targeted PCR amplification. The sequencing was performed using a PE150 strategy on the Illumina Novaseq 6000.

### RNA sequencing data analysis

The initial data sets were refined using the Cutadapt (version: 1.9) tool. Subsequently, FastQC (version: 0.11.9) was employed to assess the quality of the refined data sets, specifically examine parameters such as Q20, Q30, and GC content. The HISAT2 (version: 2-2.2.1) tool was utilized to align the data sets from all samples against the human reference genome. StringTie (version: 2.1.6) and ballgown were then employed to estimate the expression levels of all transcripts, and determine the expression abundance of mRNAs quantified by calculating the FPKM (transcripts per kilobase of mapped reads per million) value. The analysis of gene differential expression between two groups was performed utilizing DESeq2 software (edgeR software is utilized in single sample and multiple group comparisons). Genes that satisfy the condition of having a false discovery rate (FDR) less than 0.05 and a fold change of 2 or more were considered as differentially expressed genes.

### qRT-PCR assay

The RNA lysis buffer was added to the cells, followed by RNA extraction using the RNA extraction kit (Beyotime, R0017S, Shanghai, CN), and subsequent determination of RNA concentration using a Quantus Fluorometer. Following that, cDNA was created with the RevertAid First Strand cDNA Synthesis Kit (Thermo, K1622, Shanghai, CN), followed by qRT-PCR using SYBR Green qPCR Master (Roche, 4943914001-SR, Shanghai, CN), and the relative level was determined using the 2^^(-ΔΔCt)^ technique. The mRNA primers used for these genes included GAS5 (5’-TATGGTGCTGGGTGCGGAT-3’, 5’-CCAATGGCTTGAGTTAGGCTT-3’), RND3 (5’- GCTCCATGTCTTCGCCAAG-3’, 5’-AAAACTGGCCGTGTAATTCTCA-3’), COMMD1 (5’-GCTACGGAGCCAGCTATATCC-3’, 5’-GGTTTGAGCAGTCAAGAATGCC-3’), TFPI2 (5’- CTGGGGCTGTCGATTCTGC-3’, 5’-TCTCCGCGTTATTTCCTGTTG-3’), IFI44L (5’-AGCCGTCAGGGATGTACTATAAC-3’, 5’-AGGGAATCATTTGGCTCTGTAGA-3’), GAPDH (5’-GGAGCGAGATCCCTCCAAAAT-3’, 5’-GGCTGTTGTCATACTTCTCATGG-3’).

### Chromatin immunoprecipitation (ChIP) detection

The ChIP assay was performed using the ChIP Assay Kit (Beyotime, P2078). In particular, 2 × 10^7^ cells were fixed with 1% formaldehyde for 10 min at room temperature, followed by quenching with 125 mM glycine for 5 min. After lysis, cell nuclei were pelleted by centrifugation at 1000 rpm for 5 min at 4°The chromatin was fragmented through sonication to achieve an average DNA fragment size of 200–500 base pairs, and then incubated with the following antibodies: (IgG, Abcam, ab216352, 1:30, Hangzhou, CN; SIRT7, Abcam, ab259968, 1:50；H3K18ac, Abcam, ab40888, 1:50；EZH2, Abcam, ab307646, 1:50；H3K27me^3^, Abcam, ab6002, 1:50) at 4 °C overnight for chromatin immunoprecipitation. The DNA was extracted using the phenol-chloroform method and subjected to qPCR.

### Co-immunoprecipitation (CO-IP) assays

The cell culture medium was replaced with an appropriate amount of IP lysis buffer. After a 15-min incubation, the mixture was centrifuged to collect the supernatant for protein extraction and quantification. 500 µg of protein was combined with 500 µl of chilled PBS. Agarose Protein A + G beads were thoroughly mixed and centrifuged at 3000 rpm for 5 min. The beads were divided into two portions: one for non-specific binding and the other for antibody binding. The sample was pre-cleared by adding 30 µl of 50% Agarose Protein A + G to each tube and rotating at 4 °C for 2 h. After centrifugation at 4 °C and 3000 rpm for 5 min, the supernatant was transferred to a new tube to remove the protein A + G beads. IP antibodies (SIRT7, Abcam, ab259968, 1:50; EZH2, Abcam, ab307646, 1:50) were added to 500 µl of total protein and incubated overnight at 4 °C. 30 µl of 50% Agarose protein A + G was added to each tube and incubated for 6 h at 4 °C on a shaker. The mixture was centrifuged at 3000 rpm for 5 min, the pellet was collected, resuspended in a buffer solution, boiled for 5 min, and then centrifuged at 12000 rpm for 10 min after cooling to room temperature. Finally, 30 µl of the supernatant was used for Western blot detection.

### IP assays

Cells were incubated with sufficient cell lysis buffer, then centrifuged at 12000 g for 30 min to obtain the supernatant. Next, 1 μg of specific antibody (EZH2, Abcam, ab307646, diluted 1:50) and 30 μL protein A/G beads were added to the lysate. Beads were pelleted by centrifugation, washed thrice, and resuspended in 15 μL of buffer. The mixture was boiled for 10 min before being subjected to Western blot analysis.

### Western blot

Cells were lysed with RIPA lysate (Beyotime, P0013B) to obtain the proteins. The concentration of the protein was determined using the BCA protein assay kit (Beyotime, P0012S). For each group, *n* = 3. Subsequently, protein samples were separated via SDS-PAGE and transferred onto PVDF membranes. The PVDF membranes were immersed in TBST supplemented with 5% skim milk powder and incubated at room temperature for 2 h. The blocking solution was mixed with specific primary antibodies (SIRT7, Abcam, ab259968, 1:1000; EZH2, Abcam, ab191250, 1:1000; RND3, Abcam, ab171799, 1:1000; Acetyllysine, Abcam, ab21623, 1:50; Succinyllysine, PTM, PTM-419, 1:50, Hangzhou, CN; GAPDH, Abcam, ab8245, 1:1000), and the PVDF membranes were exposed to the primary antibody incubation solution and left to incubate overnight at 4 °C. This was followed by washing with TBST, and the PVDF membranes were soaked in HRP-labeled secondary antibody incubation solution (HRP-labeled goat anti-rabbit secondary antibody, Boster, BA1054, 1:10000, Wuhan, CN) and incubated for 2 h at 37 °C. The working solution in the ECL kit (Beyotime, P0018) was added to the PVDF membranes. It was allowed to react for few minutes, and the excess substrate solution was removed with filter paper and photographed using a developing instrument. The Western blot results were analyzed using Image J software (National Institutes of Health, USA). Original data for the Western blots are reported as WB Original Data.

### Animal tumorigenesis

The male Balb/c nude mice aged 7 weeks were obtained from Hubei Biont Biotechnology Co, Ltd. The mice were housed in a room with a 12-hour light/dark cycle and a temperature maintained at 24 ± 0.5°C. Twenty-five Balb/c nude mice were randomly allocated into 5 groups, each with 5 mice. A total of 2 × 10^6^ UMUC3 cells in 100 µL PBS were injected into the right axilla of the mice. Fourteen days post-inoculation, intratumoral injections of lentiviruses containing NC shRNA, SIRT7 shRNA, and/or EZH2 shRNA lentivirus were administered, together with intraperitoneal injections of CDDP (3 mg/kg) or PBS. Nude mice were fed consistently, and their body weight, tumor length, and width were measured every 3 days. Tumor volume was calculated using the formula: tumor volume = 1/2 × length × width^^2^. On day 28 post-inoculation, the mice were euthanized via cervical dislocation. Subcutaneous tumors were excised, rinsed with PBS, photographed, and measured for size and weight. Some samples were fixed in 4% paraformaldehyde for histological analysis, while others were stored in liquid nitrogen for RNA extraction and molecular studies, including gene expression analysis.

### Statistical analysis

Statistical analysis and visualization were performed using the R software and SPSS 27.0 statistical software. Count data were expressed as frequency (composition ratio, rate), and inter-group comparisons were assessed using the χ^2^ test. Quantitative data were presented as mean ± SD, with inter-group comparisons between two groups analyzed using independent sample t-tests. For comparisons involving more than two groups, the least significant difference (LSD) method in one-way analysis of variance (one-way ANOVA) were employed. The Kaplan-Meier survival curve function was employed to analyze and compare the OS and DFS of patients with varying levels of SIRT7 and RND3 expression. Survival curves were constructed and the difference in survival rates was compared with the log-rank test to determine if there was statistical significance. The clinical pathological features and levels of SIRT7 and RND3 expression in patients were incorporated into the COX proportional hazards regression model for both univariate and multivariate analysis in order to examine factors influencing patient prognosis. In a two-tailed test at a significance level of α = 0.05, any statistical analyses yielding *P* < 0.05 were considered statistically significant.

## Results

### The sensitivity of BCa patients to platinum-based treatments correlated with SIRT7 expression in cancerous tissues

**Fig. 1:**
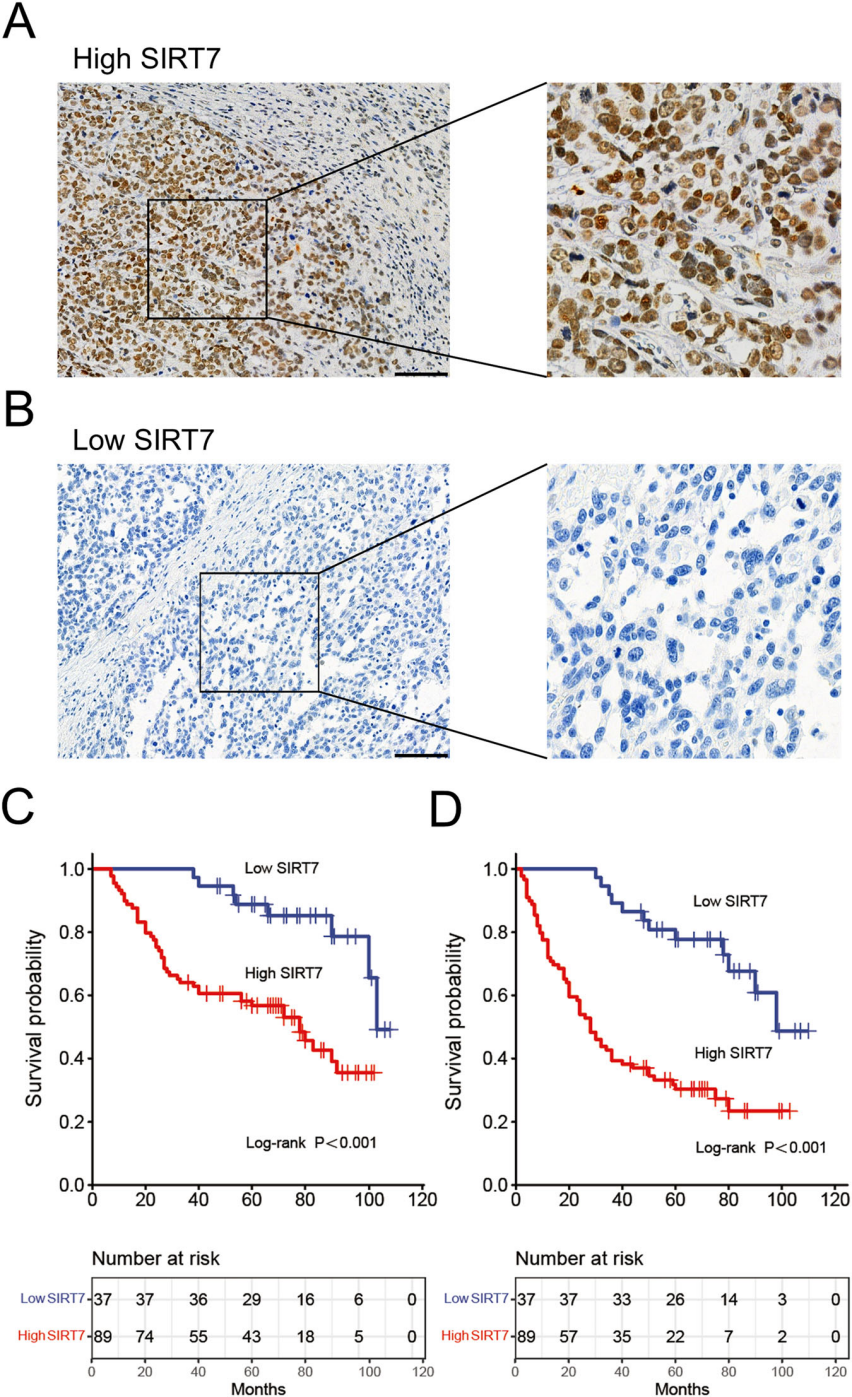
The association between the expression levels of SIRT7 in bladder cancer tissues and the efficacy of postoperative chemotherapy. **A**, **B** Representative images of IHC detection of the expression of SIRT7 in bladder cancer tissues. **A** The IHC images of high SIRT7 expression in bladder cancer tissues. **B** IHC images of low SIRT7 expression in bladder cancer tissues. **C** Kaplan-Meier curve analysis comparing the OS of the high and low SIRT7 content groups. **D** Kaplan-Meier curve analysis comparing DFS between high and low SIRT7 expression groups. A cohort of 126 bladder cancer patients received resection and platinum-based chemotherapy in the study, with their surgical specimens and survival data were examined. Statistical analysis and plotting were performed using the R software. The Kaplan-Meier curve function was employed to compare OS and DFS between patients with high SIRT7 expression and those with low SIRT7 level, with survival curves were constructed. The discrepancy in survival rates was analyzed using the log-rank test to determine significant differences. Statistical significance was defined as a *p* < 0.05. Scale bar in (**A**) and (**B**) is 100 μm.

This study involved 126 Muscle-Invasive Bladder Cancer (MIBC) patients who underwent platinum-based chemotherapy post-surgery. These patients’ cancer tissues were surgically resected and examined. Among these patients, 105 were male and 70 were aged ≥ 63 years. Of the included patients, 119 and 7 received the CDDP+gemcitabine and carboplatin+gemcitabine combination therapies, respectively. Furthermore, 29 and 62 cases exhibited Lymph Node Metastasis (LNM; N1, N2) and vascular invasion, respectively. Moreover, 19 cases presented with various histological variations, including adenoid and squamous differentiations, lymphoepithelioma-like features, and plasma cell-like morphology. Immunohistochemistry (IHC) analysis was used to assess SIRT7 protein levels in each surgically resected BCa tissue sample (Fig. 1A, B). According to the results, 89 tissue samples exhibited SIRT7 upregulation. Furthermore, compared to the low SIRT7 content group, the high SIRT7 content group exhibited significantly higher T3/T4 and N1/N2 proportions and vascular invasion levels. Conversely, the two groups showed no significant differences in age, gender, Body Mass Index (BMI), smoking history, chemotherapy regimen, and histological variations (Table 1).

**Table 1:** The correlation between the expression levels of SIRT7 and RND3 and the clinical features of bladder cancer.

Characteristics	Number of patients	SIRT7 (low)	SIRT7 (high)	*P* value *of SIRT7*	RND3 (low)	RND3 (high)	*P* value *of RND3*
**Patients**	126	37 (29.4)	89 (70.6)		85 (67.5)	41 (32.5)	
**Age (year)**				0.54			0.395
<63	56	18(48.6)	38(42.7)		40(47.1)	16(39.0)	
≥ 63	70	19(51.4)	51(57.3)		45(52.9)	25(61.0)	
**Gender**				0.93			0.061
Male	105	31(83.8)	74(83.1)		75(88.2)	30(73.2)	
Female	21	6(16.2)	15(16.9)		10(11.8)	11(26.8)	
**BMI (kg/m**^**2**^**)**				0.443			0.808
<24	48	16(43.2)	32(36.0)		33(38.8)	15(36.6)	
≥24	78	21(56.8)	57(64.0)		52(61.2)	26(63.4)	
**Smoking history**				0.311			0.99
Yes	80	21(56.8)	59(66.3)		54(63.5)	26(63.4)	
No	46	16(43.2)	30(33.7)		31(36.5)	15(36.6)	
**Chemotherapy protocol**				0.962			0.818
Cisplatin and Gemcitabine	119	35(94.6)	84(94.4)		80(94.1)	39(95.1)	
Carboplatin and Gemcitabine	7	2(5.4)	5(5.6)		5(5.9)	2(4.9)	
**T stage**				<0.001			0.106
T2	70	29(78.4)	41(46.1)		43(50.6)	27(65.9)	
T3/T4	56	8(21.6)	48(53.9)		42(49.4)	14(34.1)	
**Lymph node metastasis**				0.01			0.516
N0 / Nx	97	34(91.9)	63(70.8)		64(75.3)	33(80.5)	
N1 /N2	29	3(8.1)	26(29.2)		21(24.7)	8(19.5)	
**Vascular invasion**				<0.001			0.006
No	64	31(83.8)	33(37.1)		36(42.4)	28(68.3)	
Yes	62	6(16.2)	56(62.9)		49(57.6)	13(31.7)	
**Histological variant**				0.388			0.664
Pure urothelial	107	33(89.2)	74(83.1)		73(85.9)	34(82.9)	
Variant histology	19	4 (10.9)	15(16.9)		12(14.1)	7(17.1)	

Additionally, according to the Kaplan-Meier (K-M) survival curve analysis results, the low SIRT7 content group showed significantly higher Disease-Free Survival (DFS) and OS rates than the high SIRT7 content group (Fig. 1C, D). Furthermore, univariate COX regression model analysis showed that T stage, LNM, vascular invasion, and SIRT7 content correlated significantly with both the DFS and OS rates (Tables 2–3). On the other hand, SIRT7 content correlated with only DFS, according to the multivariate COX regression analysis results (Table 4). These findings collectively suggest a potential relationship between SIRT7 content and BCa progression and sensitivity to platinum-based treatments.

**Table 2:** Univariate Cox regression analysis was conducted to examine the correlation between SIRT7 and RND3 expression and the OS of patients with bladder cancer.

Characteristics	OS	
	HR (95%CI)	*P* value
**Age (year)**		
≥63 VS <63	1.389(0.806–2.393)	0.237
**Gender**		
Male VS Female	1.806(0.771–4.230)	0.173
**BMI (kg/m**^**2**^**)**		
≥24 VS <24	1.293(0.753–2.220)	0.351
**Smoking history**		
Yes VS No	1.224(0.692–2.165)	0.448
**T stage**		
T3/T4 VS T2	5.628(2.974–10.651)	<0.001
**Lymph node metastasis**		
N1/N2 VS N0/Nx	4.297(2.441–7.556)	<0.001
**Vascular invasion**		
Yes VS No	4.485(2.393–8.405)	<0.001
**Histological variant**		
Yes VS No	1.510(0.733–3.109)	0.263
**Chemotherapy protocol**		
Cisplatin VS Carboplatin	1.626(0.395–6.682)	0.501
**SIRT7 expression**		
High VS Low	4.027(1.808–8.966)	<0.001
**RND3 expression**		
High VS Low	0.327(0.154–0.696)	0.004

**Table 3:** Univariate Cox regression analysis was conducted to examine the correlation between SIRT7 and RND3 expression and the DFS of patients with bladder cancer.

Characteristics	DFS	
	HR (95%CI)	*P* value
**Age (year)**		
≥63 VS <63	1.119(0.710–1.764)	0.628
**Gender**		
Male VS Female	1.271(0.898–2.082)	0.108
**BMI (kg/m**^**2**^**)**		
≥24 VS <24	1.151(0.725–1.829)	0.550
**Smoking history**		
Yes VS No	1.201(0.745–1.936)	0.451
**T stage**		
T3/T4 VS T2	3.987 (2.415–6.584)	<0.001
**Lymph node metastasis**		
N1/N2 VS N0/Nx	3.199 (1.968–5.199)	<0.001
**Vascular invasion**		
Yes VS No	3.099 (2.009–4.780)	<0.001
**Histological variant**		
Yes VS No	1.176 (0.619–2.234)	0.620
**Chemotherapy protocol**		
Cisplatin VS Carboplatin	0.789(0.318–1.956)	0.608
**SIRT7 expression**		
High VS Low	4.080(2.719–7.641)	<0.001
**RND3 expression**		
High VS Low	0.433(0.240–0.783)	0.006

**Table 4:** Multivariate Cox regression analysis was conducted to examine the correlation between SIRT7 and RND3 expression and both OS and DFS of patients with bladder cancer.

Characteristics	OS		DFS	
	HR (95%CI)	*P* value	HR (95%CI)	*P* value
**T stage**				
T3/T4 VS T2	3.270(1.570–6.810)	0.002	2.654(1.473–4.780)	0.001
**Lymph node metastasis**				
N1/N2 VS N0/Nx	2.002(1.042–3.844)	0.037	2.529(1.401–4.556)	0.002
**Vascular invasion**				
Yes VS No	1.347 (0.582–3.133)	0.486	0.974(0.504–1.885)	0.939
**SIRT7 expression**				
High VS Low	2.324 (0.993–5.439)	0.052	3.281(1.709–6.302)	<0.001
**RND3 expression**				
High VS Low	0.424 (0.197–0.915)	0.029	0.539(0.293–0.992)	0.047

This study also examined data from 117 BCa patients who did not receive platinum-based chemotherapy either pre- or post-surgery. Notably, the data was retrieved from The Cancer Genome Atlas (TCGA) database. According to the results, SIRT7 content in these BCa tissues did not correlate significantly with patients’ DFS rates (Fig. S1), highlighting the specificity of SIRT7 in the efficacy of platinum-based adjuvant chemotherapy.

### SIRT7 decreased BCa cell CDDP sensitivity

**Fig. 2:**
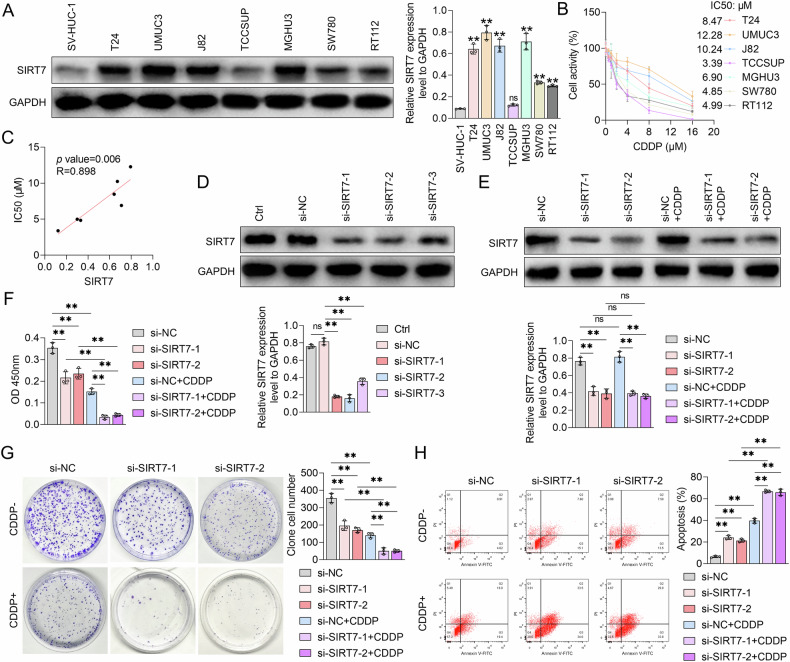
The effect of SIRT7 siRNA on the proliferation and cisplatin responsiveness of UMUC3 cells. **A** Western blot analysis showing the expression levels of SIRT7 protein in SV-HUC-1 cells and various bladder cancer cell lines, GAPDH served as the internal control, and quantitative analyses are shown on the right. **B** CCK-8 assay was utilized to determine the effect of various concentrations of CDDP on the viability of different bladder cancer cell lines, and the IC50 value was determined. **C** Pearson correlation coefficient was calculated to assess the relationship between the IC50 values of CDDP and intracellular SIRT7 protein levels in bladder cancer cell lines. **D** Western Blot analysis was to determine the impact of NC siRNA or SIRT7 siRNA transfection on the intracellular SIRT7 protein levels in UMUC3 cells. The quantification graph of protein band is shown. **E** Western Blot analysis was conducted to evaluate the influence of SIRT7 siRNA and/or CDDP treatment on the intracellular SIRT7 protein content in UMUC3 cells. The graph on the right shows the expression levels of SIRT7 protein bands. **F** CCK-8 assay was used to determine the effect of SIRT7 siRNA and/or CDDP treatment on the activity of UMUC3 cells. **G** Plate colony assay results showing the impact of SIRT7 siRNA, CDDP, and their combination on the colony formation ability of UMUC3 cells. The number of colony cells is shown on the right. **H** Flow cytometry analysis of the apoptosis level of UMUC3 cells after treatment with NC siRNA or SIRT7 siRNA in combination with or without CDDP. The quantitative analysis graph is shown on the right. Quantitative data are presented as mean ± SD, *n* = 3. One-way ANOVA was used to assess group differences, with ns indicating *p* > 0.05 and ** indicating *p* < 0.01.

To explore the regulatory role of SIRT7 in BCa cell CDDP sensitivity, we assessed SIRT7 protein content in the human ureteral epithelial immortalized cell line SV-HUC-1, as well as in various BCa cell lines, using Western Blot (WB) analysis. According to the results, except TCCSUP cells, all other BCa cell lines exhibited significantly higher SIRT7 protein levels than SV-HUC-1 cells (Fig. 2A). Among the BCa cell lines with higher SIRT7 levels than SV-HUC-1 cells, the UMUC3 cell line had the highest SIRT7 protein content, followed sequentially by the T24, J82, MGHU3, SW780, and RT112 cell lines (Fig. 2A). Subsequently, the sensitivity of these BCa cell lines to CDDP was assessed using the CCK-8 assay. According to the results, compared to other BCa cell lines, UMUC3 and J82 cell lines had higher IC50 values for CDDP (Fig. 2B). Furthermore, correlation analyses revealed that the SIRT7 protein content correlated significantly positively with the IC50 value for CDDP in BCa cell lines (Fig. 2C), implying a strong relationship between SIRT7 expression and BCa cell CDDP sensitivity.

To further explore the role of SIRT7 in cell growth and CDDP sensitivity in UMUC3 cells, we first constructed SIRT7 siRNA, which effectively downregulated SIRT7 in these cells (Fig. 2D). Following that, UMUC3 cells were treated with NC siRNA or SIRT7 siRNA, with or without CDDP. According to the WB analysis results, CDDP treatment did not significantly alter the intracellular SIRT7 protein levels in UMUC3 cells (Fig. 2E). Furthermore, the CCK-8 assay results demonstrated a significantly lower UMUC3 cell viability in the SIRT7 siRNA treatment group compared to the NC siRNA control group that lacked CDDP (Fig. 2F). Notably, the plate colony formation assay confirmed these results, revealing a substantial decrease in the number of cell colonies in UMUC3 cells following SIRT7 siRNA treatment in the absence of CDDP (Fig. 2G). Additionally, SIRT7 knockdown induced UMUC3 cell apoptosis in CDDP absence (Fig. 2H), and facilitated CDDP-mediated effects, including reduced cell viability, colony formation inhibition, and increased apoptosis in UMUC3 cells (Fig. 2F-H). These findings collectively suggest that SIRT7 may promote BCa cell growth and CDDP resistance.

### SIRT7 suppressed RND3 expression in BCa cells by decreasing H3K18 acetylation

**Fig. 3:**
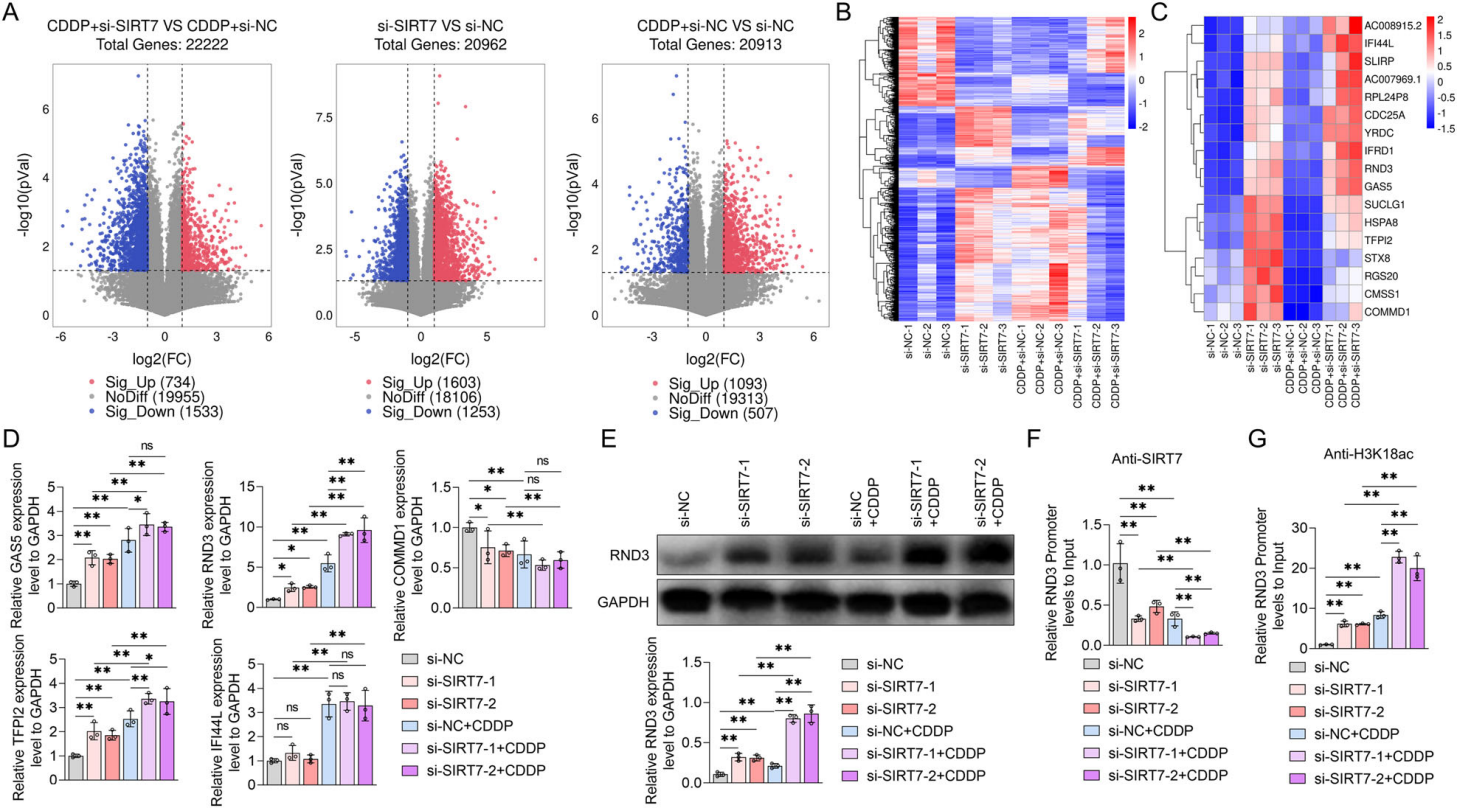
The effects of SIRT7 siRNA and CDDP on the modulation of RND3 expression and H3K18ac levels within the promoter region of RND3 in UMUC3 cells. **A**–**C** UMUC3 cells were transfected with either NC siRNA or SIRT7 siRNA, and treated with or without CDDP. The RNA expression profiles of each group of cells were subjected to RNA sequencing. **A** Volcano plot of differentially expressed genes between two treatment groups of UMUC3 cells. **B** Heatmap of differentially expressed genes in different treatment groups of UMUC3 cells. **C** Heatmap of differentially expressed genes that met the screening criteria. The screening criteria include genes that were upregulated in UMUC3 cells treated with SIRT7 siRNA compared to those with NC siRNA in the absence of CDDP, with a fold change (FC) exceeding 2; fold change of gene expression in SIRT7 siRNA vs that in NC siRNA in UMUC3 cells treated without CDDP > 1; the consistency of gene expression changes following treatment with CDDP in UMUC3 cells treated with NC siRNA should align with the gene expression changes induced by CDDP in cells treated with SIRT7 siRNA. **D** MRNA level of tumor suppressor genes from (**C**) in UMUC3 cells treated with NC siRNA or SIRT7 siRNA together with or without CDDP was measured by qRT-PCR. **E**–**G** After transfection with either NC siRNA or SIRT7 siRNA, UMUC3 cells were exposed to CDDP. **E** The protein expression levels of RND3 were by Western Blot, GAPDH served as the internal control, and the quantification of protein bands is shown by the graph below the bands. **F**–**G** ChIP-PCR experiments were used to analyze the enrichment content of SIRT7 in the promoter region of RND3 (**F**) and the level of H3K18ac (**G**) in UMUC3 cells with different treatment. Quantitative data are presented as mean ± SD, *n* = 3. One-way ANOVA was used to assess group differences, with ns indicating *p* > 0.05, * indicating *p* < 0.05 and ** indicating *p* < 0.01.

To elucidate the molecular mechanisms through which SIRT7 modulates BCa cell CDDP sensitivity, we subjected RNA samples from UMUC3 cells treated with SIRT7 siRNA or NC siRNA, with or without CDDP, to RNA sequencing. Since SIRT7 can inhibit histone acetylation levels and silence gene expression [12], we focused on genes that were up-regulated in UMUC3 cells following SIRT7 knockdown. According to the results, compared to the NC siRNA and CDDP groups, UMUC3 cells treated with SIRT7 siRNA and CDDP exhibited 734 Differentially Expressed Genes (DEGs) (Fig. 3A). After SIRT7 knockdown, 199 genes were significantly upregulated and 212 genes were significantly downregulated in UMUC3 cells relative to the cells treated with NC siRNA alone. Notably, the expression trends of these genes were similar in UMUC3 cells treated with CDDP and si-SIRT7 and those treated with CDDP and si-NC (Fig. 3B). These DEGs were further subjected to Gene Ontology (GO) and Kyoto Encyclopedia of Genes and Genomes (KEGG) enrichment analyses. According to the results, these DEGs were involved in ribosome biogenesis and extracellular structure organization, among other Biological Processes (BPs) (Fig. S2A and S2B). Furthermore, the DEGs were implicated in several pathways, including RNA polymerase activity and ECM-receptor interaction, which were disturbed by SIRT7 knockdown in UMUC3 cells (Fig. S2C and S2D). These findings suggest that SIRT7 can affect both the BPs and pathways in BCa cells.

Subsequently, we screened out 17 upregulated genes that had similar expression patterns in CDDP-treated UMUC3 cells and in cells treated with NC siRNA or SIRT7 siRNA without CDDP (Fig. 3C). The mRNA levels of several tumor suppressor genes were then identified using the qRT-PCR assay. According to the results, SIRT7 knockdown significantly upregulated the RND3 and TFPI2 mRNA levels in UMUC3 cells in the presence or absence of CDDP treatment, with RND3 expression exhibiting a more pronounced increase (Fig. 3D). Furthermore, CDDP treatment alone resulted in RND3 mRNA upregulation in UMUC3 cells (Fig. 3D). Notably, WB analysis confirmed these results, revealing that SIRT7 knockdown and CDDP stimulation significantly upregulated the RND3 protein levels in UMUC3 cells. Moreover, SIRT7 knockdown potentiated the CDDP treatment-induced RND3 protein upregulation (Fig. 3E).

Subsequently, we assessed SIRT7 enrichment in the RND3 gene promoter region using ChIP-qPCR experiments and our findings indicated a significant SIRT7 protein presence in this region. Notably, SIRT7 knockdown and CDDP stimulation decreased the SIRT7 protein enrichment levels in the RND3 gene promoter region. Furthermore, SIRT7 knockdown exacerbated SIRT7 protein downregulation in the RND3 gene promoter region in CDDP-treated UMUC3 cells (Fig. 3F). Subsequently, we assessed H3K18 acetylation levels within the RND3 gene promoter region and identified H3K18ac presence in this region. Notably, both SIRT7 knockdown and CDDP stimulation upregulated H3K1ac. Moreover, SIRT7 siRNA treatment enhanced the promotional effect of CDDP on H3K18 acetylation within the RND3 gene promoter region in UMUC3 cells (Fig. 3G). These findings suggests that SIRT7 can bind to the RND3 gene promoter region, leading to H3K18ac and RND3 downregulation in BCa cells. Overall CDDP can obstruct the binding of SIRT7 to the RND3 gene promoter region, promoting RND3 expression.

### RND3 correlated with the therapeutic effects of platinum-based treatments and SIRT7 expression in BCa patients

**Fig. 4:**
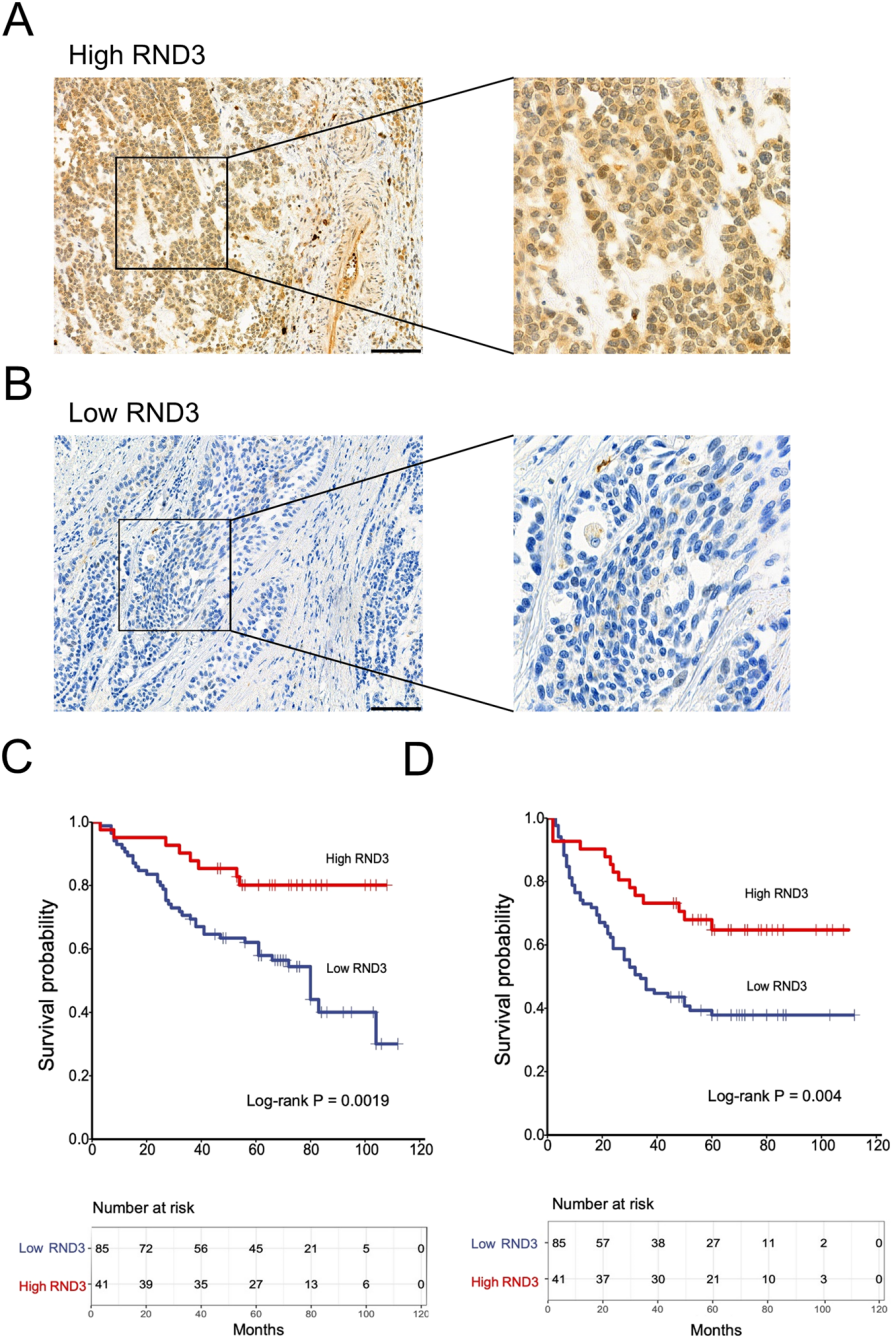
The association between the expression levels of RND3 in bladder cancer tissues and the efficacy of postoperative chemotherapy. **A**, **B** Representative images of IHC detection of the expression level of RND3 in bladder cancer tissues. **A** IHC images of high RND3 expression level in bladder cancer tissues. **B** IHC images of low RND3 expression level in bladder cancer tissues. **C** Kaplan-Meier curve analysis comparing the OS between high and low RND3 expression groups. **D** Kaplan-Meier curve analysis comparing DFS between high and low RND3 content groups. A cohort of 126 bladder cancer patients who underwent resection and received platinum-based chemotherapy was included in the study, with their surgical specimens and survival data being examined. Statistical analysis and plotting were performed using the R software. The Kaplan-Meier curve function was employed to compare the OS and DFS between patients with high RND3 expression and those with low RND3 level, and survival curves were constructed. Differences in survival rates were evaluated using the log-rank test to determine significant differences. Statistical significance was defined as a *p* < 0.05. Scale bar in (**A**) and (**B**) is 100 μm.

The same cohort from which the IHC samples were retrieved was also assessed for RND3 expression using the IHC assay (Fig. 4A, B). According to the results, 85 tissue samples exhibited low RND3 expression. Furthermore, compared to the high RND3 expression group, the low RND3 expression group showed significantly higher vascular invasion levels. Conversely, the two groups showed no significant differences in age, gender, BMI, smoking history, chemotherapy protocol, T stage, LNM, and histological variation (Table 1). These findings suggest that RND3 expression correlated with BCa metastasis.

The correlation of RND3 with both the DFS and OS rates in BCa patients treated with platinum-based adjuvant chemotherapy was assessed using the K-M curve survival analysis. According to the results, patients in the high RND3 expression group exhibited significantly higher DFS and OS rates than those in the low RND3 expression group (Fig. 4C, D). Furthermore, univariate COX regression model analysis showed that RND3 expression correlated significantly with both the DFS and OS rates (Tables 2–3). Multivariate COX regression analysis also revealed that RND3 expression correlated with both the DFS and OS rates (Table 4). These findings suggest that RND3 expression correlated with the sensitivity of BCa patients to platinum-based treatments.

Spearman’s correlation analysis was also conducted to explore the SIRT7-RND3 association. According to the results, RND3 expression correlated significantly negatively with SIRT7 content in BCa tissues (Table 5), implying that SIRT7 can regulate RND3 expression in BCa.

**Table 5:** Spearman correlation analysis of SIRT7 and RND3 expression with bladder cancer.

Features	*N*	RS	*P* value
SIRT7 expression / RND3 expression	126	−0.556	<0.001

### SIRT7-mediated BCa cell CDDP resistance relies on RND3

**Fig. 5:**
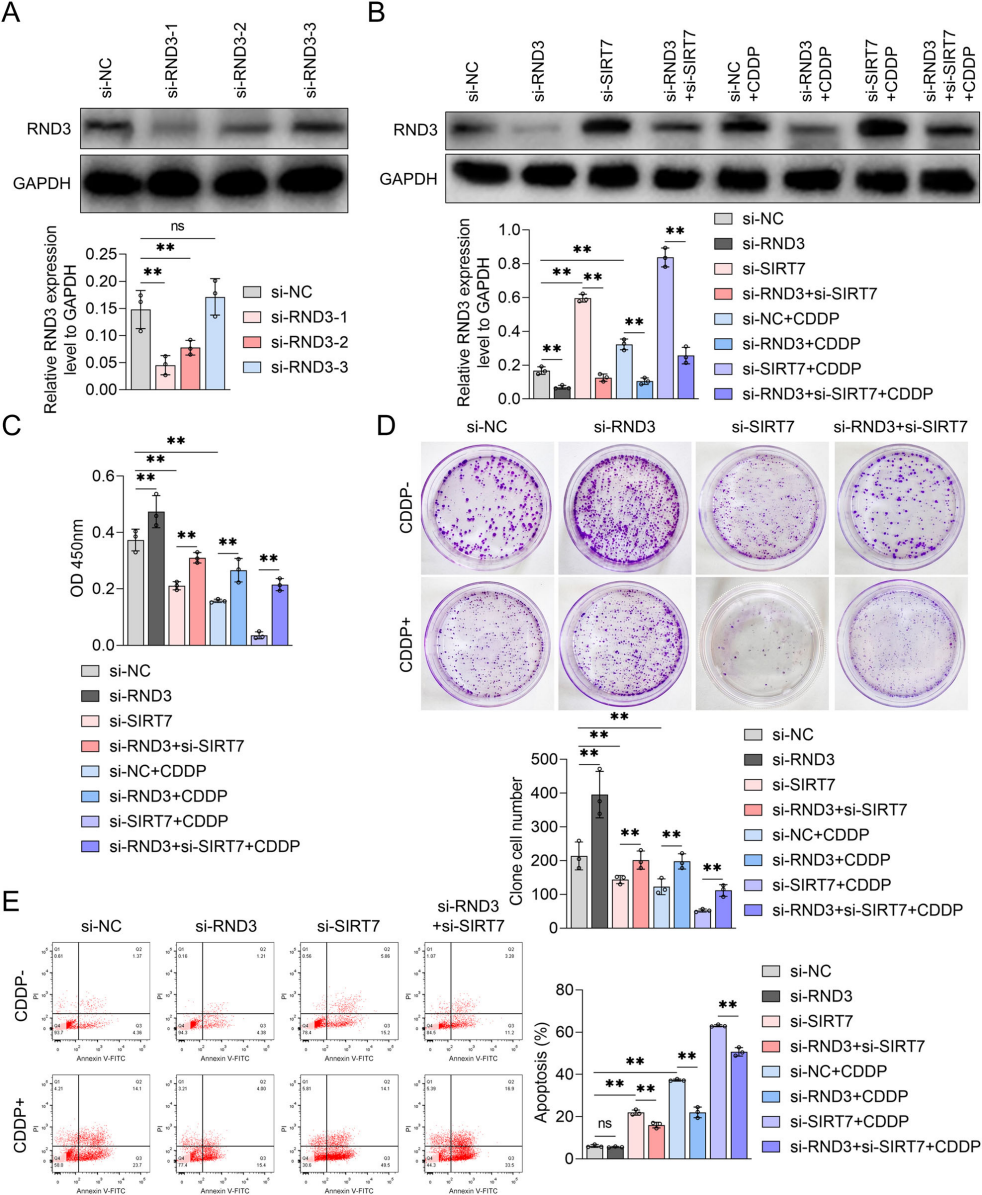
The effects of RND3 siRNA and SIRT7 siRNA on the growth and CDDP sensitivity of UMUC3 cells. **A** The expression level of RND3 protein in UMUC3 cells following transfection with either NC siRNA or RND3 siRNA was confirmed by Western Blot. The quantitative analysis of the bands is shown by the graph below the bands. **B**–**E** UMUC3 cells were transfected with NC siRNA, RND3 siRNA, or SIRT7 siRNA and treated with or without CDDP. **B** Western Blot was used to measure the RND3 protein levels, **C** CCK-8 experiments measured cell viability, **D** plate colony experiments assessed colony formation ability, and **E** flow cytometry was conducted to analyze apoptosis levels in UMUC3 cells. Quantitative data are expressed as mean ± SD, *n* = 3. One-way ANOVA was used to assess group differences, with ns indicating *p* > 0.05, and ** indicating *p* < 0.01.

To elucidate the role of RND3 in SIRT7-mediated regulation of BCa cell growth and CDDP resistance, RND3-specific siRNA was constructed, effectively downregulating RND3 protein levels in UMUC3 cells (Fig. 5A). After treating UMUC3 cells with RND3 siRNA, either alone or in combination with SIRT7 siRNA and/or CDDP, WB analysis was performed revealing that RND3 siRNA could reverse the SIRT7 knockdown-induced RND3 upregulation (Fig. 5B). Subsequently, the impact of the RND3 siRNA+SIRT7 siRNA combination treatment on UMUC3 cell proliferation was assessed using the CCK-8 and colony formation assays. According to the results, RND3 siRNA treatment significantly enhanced UMUC3 cell viability compared to NC siRNA treatment alone (Fig. 5C). Furthermore, RND3 siRNA treatment mitigated the SIRT7 knockdown-induced UMUC3 cell viability reduction (Fig. 5C). Additionally, RND3 knockdown augmented the clonogenic capacity of UMUC3 cells and counteracted the reduction in their clonogenic capacity resulting from SIRT7 siRNA treatment alone (Fig. 5D). Moreover, flow cytometry analysis demonstrated that RND3 siRNA treatment inhibited UMUC3 cell apoptosis, including SIRT7 knockdown-mediated apoptosis, in the absence of CDDP treatment (Fig. 5E). These findings collectively suggest that RND3 functions as a tumor suppressor in BCa, and that the SIRT7-facilittated BCa cell proliferation depends on RND3 downregulation.

Simultaneously, we assessed the impact of RND3 siRNA treatment on BCa cell CDPP sensitivity. According to the WB analysis results, RND3 knockdown effectively inhibited the CDDP stimulation-induced RND3 upregulation (Fig. 5B). Additionally, the CCK-8 assay results revealed that RND3 knockdown mitigated the CDDP-induced reduction in UMUC3 cell viability (Fig. 5C). The plate colony formation assay results confirmed these findings, revealing that RND3 knockdown reversed the CDDP-mediated reduction in the colony formation ability in UMUC3 cells (Fig. 5D). Furthermore, RND3 siRNA inhibited the CDDP-induced UMUC3 cell apoptosis (Fig. 5E). These findings suggest that the cytotoxic efficacy of CDDP on BCa cells is RND3 dependent, and that RND3 expression could modulate BCa cell CDDP sensitivity.

Our findings also revealed that RND3 knockdown can abrogate the promotional effect of SIRT7 siRNA on the CDDP treatment-induced reduction in UMUC3 cell viability (Fig. 5C). Additionally, RND3 knockdown mitigated the inhibitory effect of SIRT7 siRNA on the clonogenic capacity of CDDP-treated UMUC3 cells (Fig. 5D) and blocked the pro-apoptotic effect of SIRT7 siRNA in these cells (Fig. 5E). We also explored the effects of SIRT7 and RND3 overexpression on CDDP sensitivity in TCCSUP cells, which were the cells most sensitive to CDDP among the above-mentioned BCa cell lines (Fig. 2B). According to the results, RND3 overexpression not only increased the sensitivity of TCCSUP cells to CDDP, but also abolished the reversal effect of SIRT7 on CDPP-induced inhibition of cell viability and cell clone formation capacity in TCCSUP cells (Fig. S3). These findings suggest that the inhibitory role of SIRT7 in modulating BCa cell CDDP sensitivity is RND3 dependent.

### EZH2 regulated BCa cell CDDP sensitivity by upregulating H3K27me^3^ in the RND3 gene promoter region

**Fig. 6:**
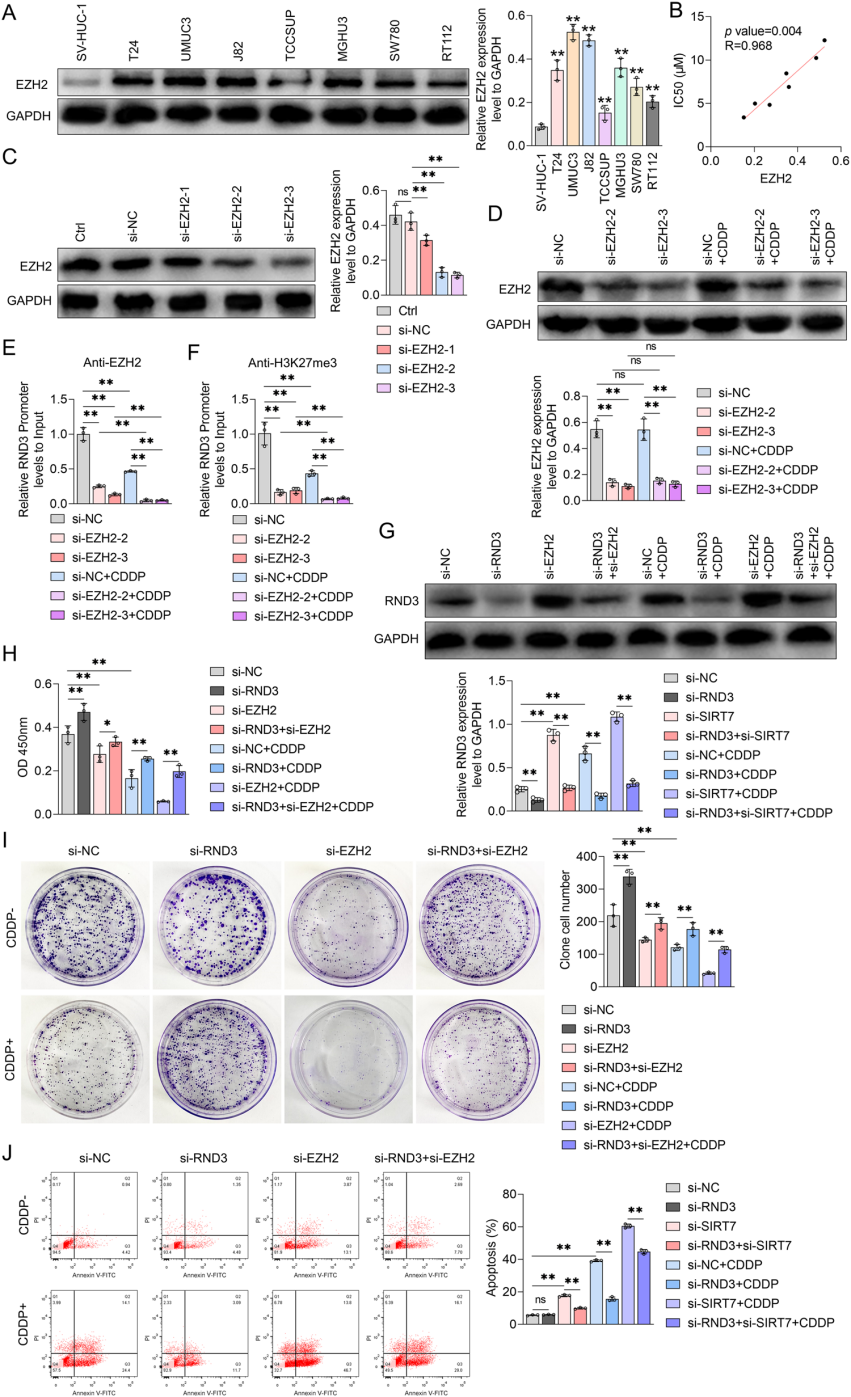
The effect of EZH2 siRNA and RND3 siRNA on the levels of H3K27me^3^ in the promoter region of RND3 and the sensitivity to CDDP in UMUC3 cells. **A** Western blot analysis was employed to assess the expression levels of EZH2 protein in SV-HUC-1 cells and various bladder cancer cell lines, GAPDH served as the internal control. Quantitative analysis of the bands is shown on the graph on the right. **B** Pearson correlation coefficient was utilized to evaluate the relationship between the IC50 values of CDDP and intracellular EZH2 protein levels in bladder cancer cell lines. **C** Western Blot analysis was performed to measure the effect of NC siRNA or EZH2 siRNA transfection on the intracellular EZH2 protein levels in UMUC3 cells. Quantitative analysis of the bands is shown by the graph on the right. **D** Western Blot analysis was conducted to evaluate the influence of NC siRNA or EZH2 siRNA treatment on the intracellular EZH2 protein expression level in UMUC3 cells following exposure to CDDP. The protein band quantification graph is below. ChIP-PCR experiments were used to analyze the enrichment content of EZH2 in the promoter region of RND3 (**E**) and the level of H3K27me^3^ (**F**) in UMUC3 cells treated with NC siRNA or EZH2 siRNA together with or without CDDP. **G**–**J** UMUC3 cells were transfected with NC siRNA, RND3 siRNA, or EZH2 siRNA and treated with or without CDDP. **G** Western Blot was used to measure the expression of RND3 proteins, **H** CCK-8 assay were performed to determine the viability of cells, **I** plate colony experiments assessed colony formation ability, and **J** flow cytometry observed apoptosis levels in UMUC3 cells. Quantitative data are presented as mean ± SD, *n* = 3. One-way ANOVA was used to assess group differences, with ns indicating *p* > 0.05, * indicating *p* < 0.05 and ** indicating *p* < 0.01.

To establish the involvement of EZH2 in BCa cell CDDP resistance, EZH2 protein levels in SV-HUC-1 cells and other BCa cell lines were assessed using WB analysis. According to the results, compared to SV-HUC-1 cells, all BCa cell lines exhibited significantly higher EZH2 protein levels, with UMUC3 and J82 cells having the highest EZH2 protein expression levels (Fig. 6A). Furthermore, Pearson’s correlation coefficient analysis revealed that the intracellular EZH2 protein content correlated significantly positively with the IC50 value for CDDP in BCa cells (Fig. 6B). These finding suggests that EZH2 expression in BCa cells may be closely associated with the cells’ sensitivity to CDDP. Subsequently, EZH2 siRNA was constructed, effectively suppressing EZH2 protein expression in UMUC3 cells (Fig. 6C). Following that, UMUC3 cells were treated with NC siRNA or EZH2 siRNA, with or without CDDP. According to the WB analysis results, CDDP did not affect EZH2 protein levels in UMUC3 cells, irrespective of whether NC siRNA or EZH2 siRNA was administered (Fig. 6D). On the other hand, the ChIP-qPCR results revealed that EZH2 bound to the RND3 gene promoter region. Furthermore, EZH2 protein abundance at this promoter region was reduced upon CDDP/EZH2 siRNA treatment (Fig. 6E), with the EZH2 siRNA+CDDP combination therapy resulting in the lowest EZH2 protein content (Fig. 6E). Additionally, both EZH2 siRNA and CDDP downregulated H3K27me^3^ levels within the RND3 gene promoter region in UMUC3 cells, with the EZH2 siRNA+CDDP combination therapy resulting in the lowest H3K27me^3^ levels (Fig. 6F). Moreover, according to the WB analysis results, EZH2 siRNA treatment significantly upregulated the RND3 protein and further enhanced the CDDP-induced RND3 expression in UMUC3 cells (Fig. 6G). This finding suggests that EZH2 can bind to the RND3 gene promoter region, elevating H3K18me^3^ levels in this region, and ultimately inhibiting RND3 expression in BCa cells. Furthermore, EZH2 could mediate the inhibitory effect of CDDP on H3K27me^3^ at the RND3 gene promoter region, as well as the subsequent promotion of RND3 transcription in BCa cells.

We further explored the impact of EZH2 on BCa cell growth and CDDP sensitivity, as well as the role of RND3 in this process, using the CCK-8 assay, colony formation assay, and flow cytometry. According to the results, EZH2 siRNA treatment alone significantly reduced the viability (Fig. 6H) and colony formation abilities (Fig. 6I) of UMUC3 cells. It also increased the UMUC3 cell apoptosis rate (Fig. 6J). These findings suggest that EZH2 could facilitate BCa cell proliferation. Furthermore, EZH2 siRNA augmented the inhibitory effects of CDDP on cell viability and colony formation abilities in UMUC3 cells, as well as the CDDP-mediated apoptosis levels in these cells (Fig. 6H-J). These findings suggest that EZH2 may decrease BCa cell CDDP sensitivity. We further explored the modulatory roles of RND3 siRNA in the effects of the EZH2 siRNA+CDDP combination treatment on RND3 protein levels in UMUC3 cells (Fig. 6G). Specifically, we examined the impact of RND3 siRNA on the EZH2 siRNA treatment-induced inhibition of BCa cell growth and enhancement of BCa cell CDDP sensitivity. According to the CCK-8 assay results, RND3 siRNA counteracted the EZH2 siRNA-induced cell viability reduction. Additionally, RND3 siRNA mitigated the CDDP-induced UMUC3 cell viability inhibition and ameliorated the decrease in cell viability resulting from the EZH2 siRNA+CDDP combination therapy (Fig. 6H). The colony formation assay confirmed these results, revealing that RND3 siRNA mitigated the suppression of colony formation abilities in UMUC3 cells resulting from EZH2 knockdown and/or CDDP treatment (Fig. 6J). Furthermore, RND3 siRNA treatment inhibited the EZH2 siRNA- and CDDP-induced apoptosis in UMUC3 cells, and counteracted the enhanced apoptosis rates in these cells resulting from the EZH2 siRNA+CDDP combination treatment (Fig. 6J). Moreover, in the absence of CDDP treatment, EZH2 overexpression increased cell viability and number of cell colonies in TCCSUP cells, a phenomenon RND3 overexpression reversed (Fig. S3). At the same time, EZH2 relieved the inhibitory effects of CDDP on cell viability and the cell clone formation capacities in TCCSUP cells, phenomena that RND3 overexpression also reversed (Fig. S3). These findings collectively suggest that EZH2 can facilitate BCa cell proliferation via RND3, potentially modulating BCa cell CDDP sensitivity in an RND3-dependent manner.

### SIRT7 modulated the EZH2-mediated H3K27me^3^ levels at the RND3 gene promoter region and inhibited RND3 expression by influencing EZH2 protein succinylation

**Fig. 7:**
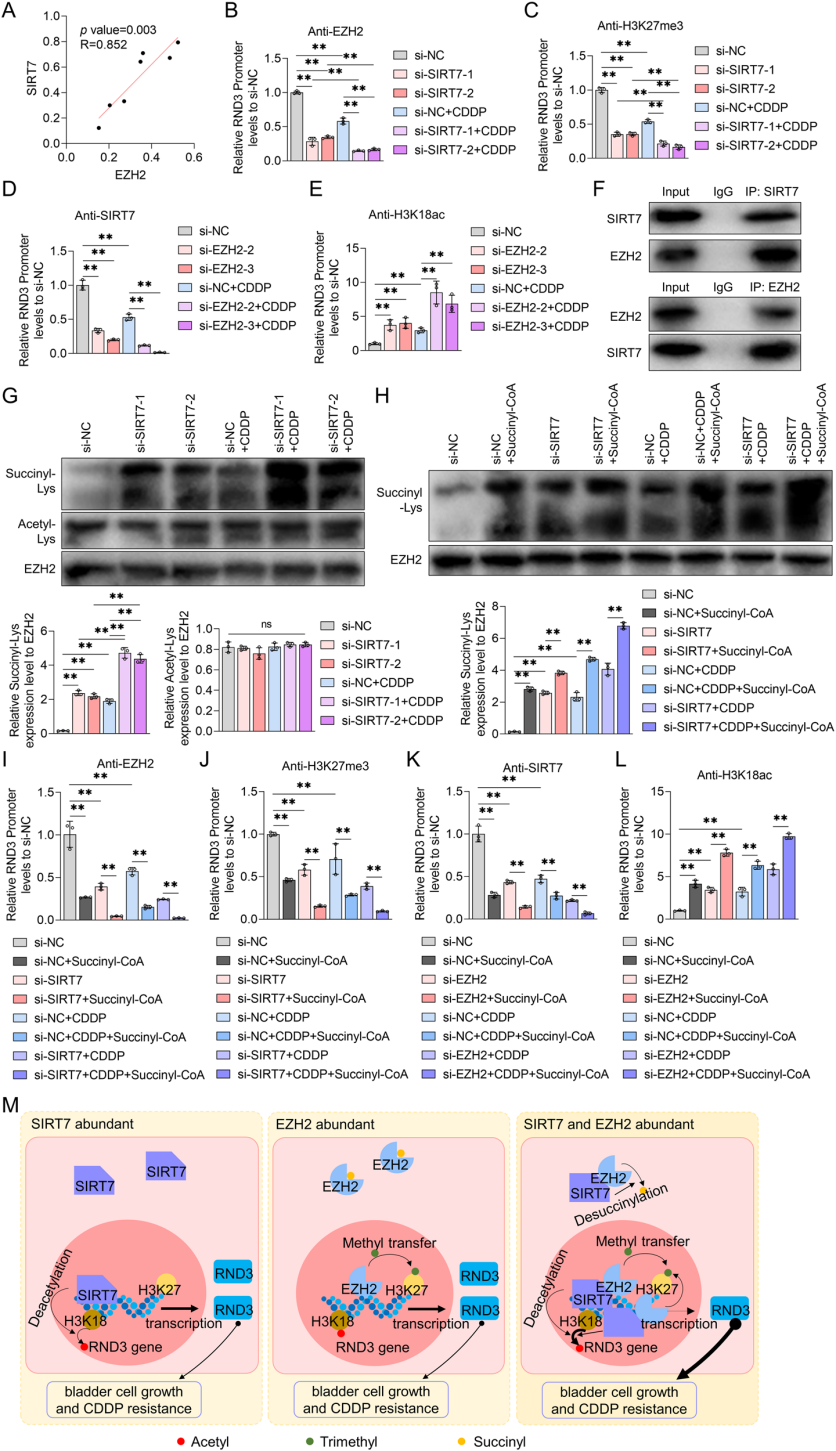
The effect of SIRT7 on the succinylation of EZH2 protein and the effect of succinylation on the recruitment of SIRT7 and EZH2 to the RND3 promoter in bladder cancer cells. **A** Pearson correlation was employed to determine the relationship between EZH2 and SIRT7 protein levels in bladder cancer cells. ChIP-PCR experiments were used to analyze the enrichment content of EZH2 in the promoter region of RND3 (**B**) and the level of H3K27me^3^ (**C**) in UMUC3 cells treated with NC siRNA or SIRT7 siRNA together with or without CDDP. ChIP-PCR experiments were used to explore the enrichment content of SIRT7 in the promoter region of RND3 (**D**) and the level of H3K18ac (**E**) in UMUC3 cells treated with NC siRNA or EZH2 siRNA together with or without CDDP. **F** CO-IP experiments were used to analyze the binding ability of SIRT7 and EZH2 in UMUC3 cells. **G** IP experiments were conducted to assess the levels of succinylation and acetylation at lysine of EZH2 protein, in UMUC3 cells subjected to transfection with NC siRNA or SIRT7 siRNA, with or without subsequent treatment with CDDP. The quantitative analyses for the diagrams are presented on the right. **H**–**J** After transfection with NC siRNA or SIRT7 siRNA, UMUC3 cells were treated with solvent or succinyl-CoA (100 μM) with or without CDDP treatment. **H** The expression of EZH2 and its lysine site succinylation level in each group of cells were analyzed by IP experiments. The band quantification diagram is shown on the right. ChIP-qPCR experiment was used to determine the enrichment of EZH2 protein (**I**) and the level of H3K27me^3^ (**J**) in the RND3 promoter region. **K**, **L** After being transfected with NC siRNA or EZH2 siRNA, UMUC3 cells were treated with solvent or succinyl-CoA (100 μM) with or without CDDP treatment. ChIP-qPCR experiment was used to investigate the enrichment of SIRT7 protein (**K**) and the level of H3K18ac (**L**) in the RND3 promoter region. **M** A schematic diagram showing the mechanism by which SIRT7 and EZH2 regulate CDDP sensitivity in bladder cancer cells through coordinated silencing of RND3 gene expression. SIRT7 interacts with EZH2, decreasing the succinylation levels of EZH2 and facilitating its recruitment to the promoter region of RND3, resulting in alterations in H3K27me^3^ content. EZH2 enhances the binding of SIRT7 to the RND3 promoter region, thereby decreasing H3K18ac expression at this promoter region and RND3 expression. This collaborative process plays a crucial role in the regulation of CDDP sensitivity in bladder cancer cells. Quantitative data are presented as mean ± SD, *n* = 3. One-way ANOVA was used to assess group differences, with ns indicating *p* > 0.05, and ** indicating *p* < 0.01.

First, we examined the correlation between the EZH2 and SIRT7 expression levels in BCa cells and found that EZH2 protein levels correlated significantly positively with SIRT7 expression (Fig. 7A). We then investigated the impact of SIRT7 siRNA on EZH2 protein enrichment within the RND3 gene promoter region in UMUC3 cells using the ChIP-qPCR assay. According to the results, compared to those treated with NC siRNA, SIRT7 siRNA-treated UMUC3 cells exhibited a lower EZH2 protein abundance in the RND3 gene promoter region. Furthermore, transfection with SIRT7 siRNA augmented the inhibitory effect of CDDP on EZH2 protein abundance in the RND3 gene promoter region (Fig. 7B). A subsequent chip-qPCR experiment demonstrated that in addition to reducing the levels of H3K27me^3^ in the RND3 gene promoter region in UMUC3 cells, SIRT7 siRNA also amplified the CDDP stimulation-induced decrease in H3K27me^3^ content in this region (Fig. 7C). Concurrently, EZH2 knockdown diminished the SIRT7 protein content in the RND3 gene promoter region and further intensified the CDDP stimulation-induced reduction in SIRT7 abundance (Fig. 7D). Furthermore, EZH2 siRNA treatment upregulated H3K18ac within the RND3 gene promoter region in UMUC3 cells, and enhanced the CDDP stimulation-mediated regultion of H3K18ac expression within the RND3 gene promoter region in these cells (Fig. 7E). These findings suggest SIRT7 and EZH2 facilitated each other’s recruitment to the RND3 gene promoter region, highlighting a reciprocal promotional mechanism between them, which led to an increase in H3K27me^3^ levels and a decrease in H3K18ac content in this region.

Subsequently, we performed Co-Immunoprecipitation (CO-IP) experiments to ascertain the potential interaction between the SIRT7 and EZH2 proteins and found that SIRT7 could bind to EZH2 (Fig. 7F). Since SIRT7 is known to modulate non-histone acetylation [21–23] and succinylation [24] levels, we proceeded to assess the alterations in the acetylation and succinylation levels of the EZH2 protein via Immunoprecipitation (IP) assays. According to the results, rather than increasing the acetylation content, SIRT7 knockdown significantly elevated the succinylation levels at the lysine residues of the EZH2 protein in UMUC3 cells (Fig. 7G). Additionally, CDDP treatment elevated the EZH2 protein succinylation levels, a regulatory effect SIRT7 siRNA further augmented (Fig. 7G). Nonetheless, SIRT7 siRNA treatment did not affect the overall EZH2 protein content (Fig. 7G). We also treated UMUC3 cells with succinyl-CoA and SIRT7 siRNA to establish the role of SIRT7 in the regulation of EZH2 protein succinylation and EZH2 recruitment at the RND3 gene promoter region. According to the IP detection results, succinyl-CoA increased the intracellular succinylation levels of the EZH2 protein and amplified the SIRT7 siRNA and/or CDDP-induced EZH2 succinylation. However, it also did not affect the overall intracellular EZH2 protein content (Fig. 7H). Following that, we explored the effects of succinyl-CoA on EZH2 protein abundance and H3K27me^3^ content in the RND3 gene promoter region in UMUC3 cells using ChIP-qPCR assays. According to the results, succinyl-CoA treatment significantly reduced both the EZH2 protein abundance and H3K27me^3^ content in the RND3 gene promoter region in UMUC3 cells. It also enhanced the inhibitory effects of SIRT7 siRNA and/or CDDP on EZH2 protein and H3K27me^3^ levels in the RND3 gene promoter region in UMUC3 cells (Fig. 7I, J). These findings suggests that SIRT7 can decrease the EZH2 protein succinylation levels and facilitate its recruitment to the RND3 gene promoter region, thus increasing the H3K27me^3^ content in this region (Fig. 6M). Furthermore, succinyl-CoA diminished the SIRT7 protein content in the RND3 gene promoter region in UMUC3 cells and upregulated H3K18ac. Additionally, it strengthened the EZH2 knockdown/CDDP-mediated SIRT7 enrichment inhibition and H3K18ac content upregulation at the RND3 gene promoter region (Fig. 7K, L). Based on these findings suggest, EZH2 protein succinylation can modulate SIRT7 recruitment to the RND3 gene promoter region (Fig. 7M).

### Suppression of SIRT7 and EZH2 expression enhanced BCa cell CDDP sensitivity in vitro

**Fig. 8:**
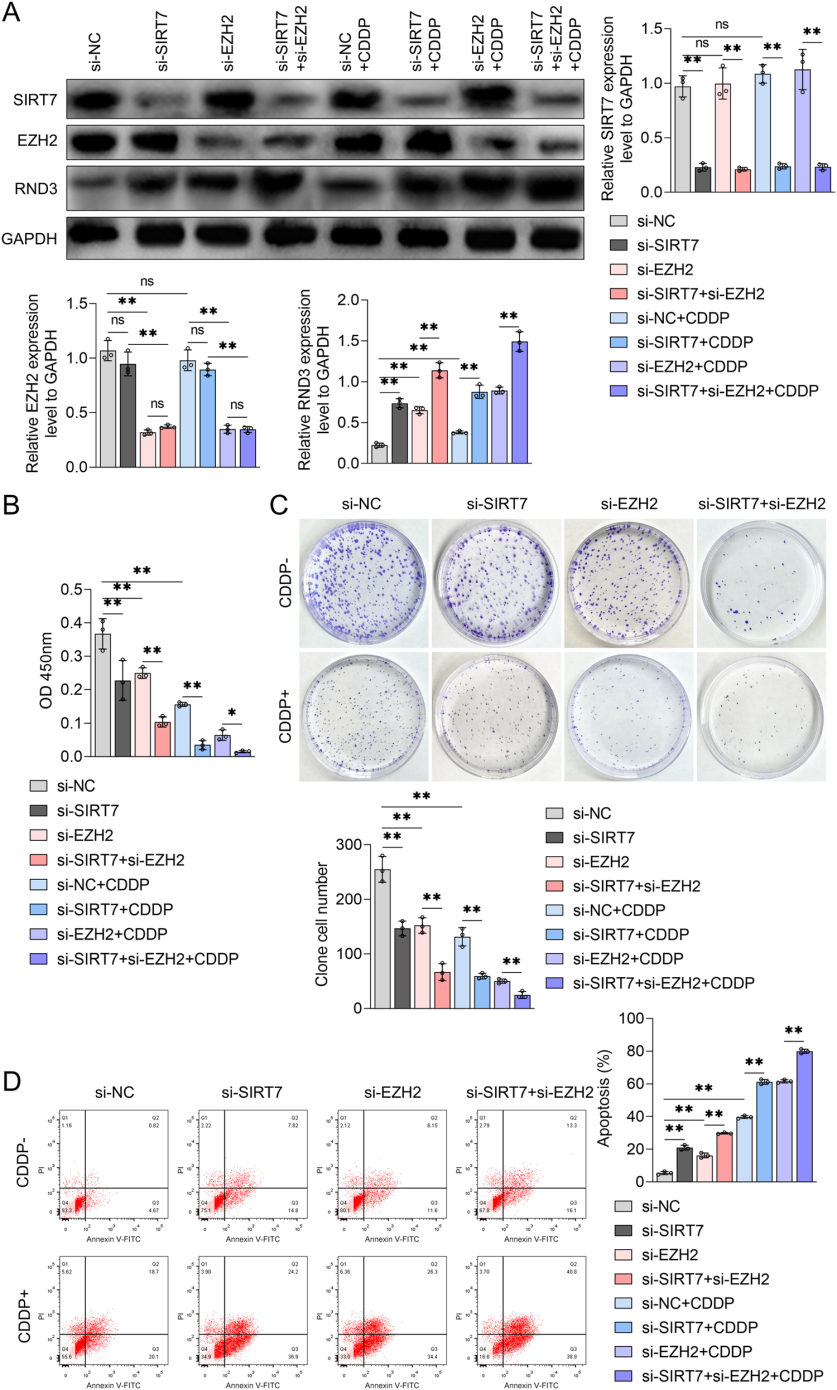
The effect of SIRT7 siRNA combined with EZH2 siRNA on the growth and CDDP sensitivity of bladder cancer cells in vitro. **A**–**D** UMUC3 cells were treated with NC siRNA or SIRT7 siRNA, followed by treatment with NC siRNA or EZH2 siRNA, and treated with either solvent or CDDP. **A** Western blot analysis was utilized to measure the levels of SIRT7, EZH2, and RND3, **B** CCK-8 assay was employed to measure cell viability, **C** plate colony experiments were employed to determine the colony formation ability of the cells, and **D** flow cytometry analysis was employed to explore apoptosis levels in UMUC3 cells. Quantitative data are expressed as mean ± SD, *n* = 3. One-way ANOVA was used to assess group differences, with ns indicating *p* > 0.05, and ** indicating *p* < 0.01.

Targeting SIRT7 and EZH2 could be a viable approach to inhibiting BCa cell proliferation and CDDP resistance. To validate this hypothesis, we treated UMUC3 cells with a combination of SIRT7 siRNA and EZH2 siRNA. According to the WB analysis results, co-inhibition of SIRT7 and EZH2 expression significantly upregulated the RND3 protein in UMUC3 cells and enhanced CDDP’s promotional effect on RND3 expression (Fig. 8A). Furthermore, EZH2 siRNA treatment did not alter the SIRT7 protein content in UMUC3 cells (Fig. 8A). According to the CCK-8 assay results, the SIRT7 siRNA+EZH2 siRNA combination therapy resulted in a significantly lower UMUC3 cell viability compared to treatment with either siRNA alone, with the combination treatment resulting in the most pronounced inhibitory effect on the viability of CDDP-stimulated UMUC3 cells (Fig. 8B). The plate clone formation assay confirmed these findings, revealing that the knockdown of both SIRT7 and EZH2 resulted in a more pronounced inhibition of the clone formation abilities of UMUC3 cells compared to SIRT7 siRNA or EZH2 siRNA treatment alone (Fig. 8C). Notably, the group that received CDDP treatment along with SIRT7 siRNA and EZH2 siRNA co-transfection exhibited the lowest number of UMUC3 cell clones (Fig. 8C). According to the flow cytometry detection results, while both SIRT7 siRNA and EZH2 siRNA treatments could induce apoptosis in UMUC3 cells when administered alone, and the combined application of the two could further enhance the level of apoptosis in these cells (Fig. 8D). Furthermore, CDDP stimulation promoted UMUC3 cell apoptosis, a regulatory function SIRT7 siRNA or EZH2 siRNA could enhance, with co-transfection of SIRT7 siRNA and EZH2 siRNA yielding the strongest promotional effect (Fig. 8D). These findings suggest that downregulating both SIRT7 and EZH2 can significantly inhibit the proliferation abilities of BCa cells and increase their sensitivity to CDDP, demonstrating a stronger regulatory function than either siRNA alone in vitro.

### Inhibiting SIRT7 and EZH2 expression increased CDDP sensitivity in BCa cells in vivo

**Fig. 9:**
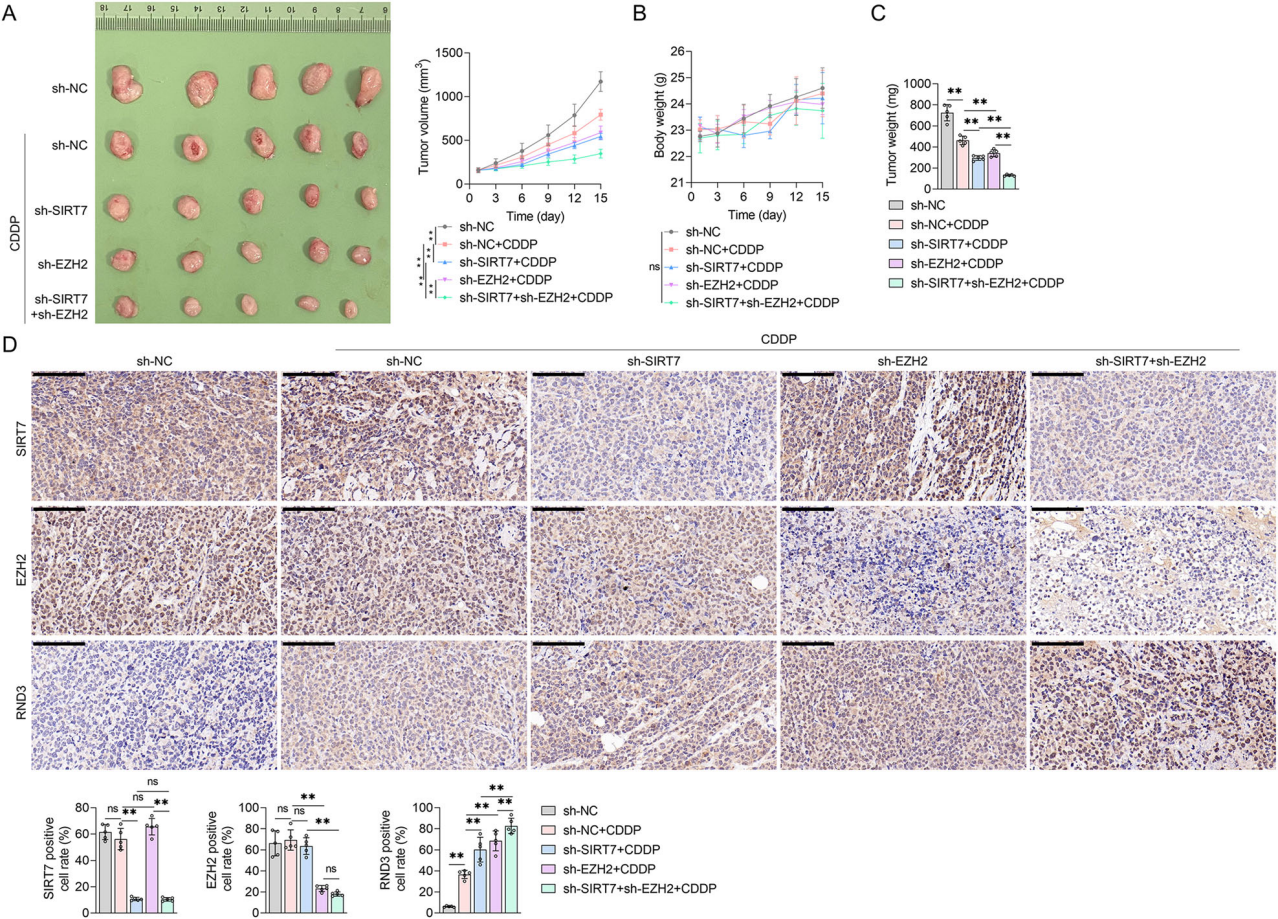
The effect of combining SIRT7 shRNA and EZH2 shRNA lentivirus on the sensitivity of bladder cancer to CDDP in vivo. UMUC3 cells were injected subcutaneously into Balb/c nude mice, and after tumor formation, NC shRNA lentivirus (sh-NC) or SIRT7 shRNA lentivirus (sh-SIRT7) and/or EZH2 shRNA lentivirus (sh-EZH2) were injected and treated with or without CDDP. **A** In vitro images of tumor tissues in mice and tumor volume graphs. **B** Body weight graphs of mice. **C** Analysis of tumor tissue weight. **D** IHC method used to detect SIRT7, EZH2, and RND3 levels in tumor tissues. The quantification analysis diagram of each indicator is presented below. Quantitative data are presented as mean ± SD, *n* = 5. One-way ANOVA was used to assess group differences, with ns indicating *p* > 0.05, and ** indicating *p* < 0.01. Scale bar in (**D**) is 100 μm.

We subcutaneously injected UMUC3 cells into mice to establish a BCa mouse model, which we used to establish the effects of inhibiting SIRT7 and EZH2 expression on BCa cell CDDP sensitivity. The animals received shRNA SIRT7 and/or shRNA EZH2 lentivirus injections, followed by CDDP treatment. The analysis of tumor photographs and tumor volume measurements revealed that CDDP treatment effectively inhibited tumor growth in the mice. Notably, the administration of the shRNA SIRT7 or shRNA EZH2 lentivirus significantly augmented this inhibitory effect. Furthermore, the administration of the shRNA SIRT7 and shRNA EZH2 lentiviruses, along with CDDP, resulted in the most pronounced tumor growth inhibition effect in mice (Fig. 9A). After the experiments, the tumor tissues were obtained and weighed, and the findings revealed that tumor weight alterations aligned with the observed tumor volume changes. These findings suggest that treatment with the shRNA SIRT7 or shRNA EZH2 lentivirus could enhance the CDDP treatment-induced reduction in tumor tissue weight. Furthermore, the concurrent administration of the shRNA SIRT7 and shRNA EZH2 lentiviruses resulted in the most significant enhancement in the CDDP treatment-induced tumor tissue weight reduction (Fig. 9C). Additionally, treatments with CDDP, as well as the shRNA SIRT7 and shRNA EZH2 lentiviruses, did not cause body weight changes in BCa-bearing mice (Fig. 9B), indicating that these treatments did not induce any substantial toxic side effects. We also assessed the SIRT7, EZH2, and RND3 protein levels in tumor tissues from each group of mice using IHC. According to the results, the shRNA SIRT7 lentivirus treatment, rather than the CDDP or shRNA EZH2 lentivirus treatment, could alter SIRT7 expression levels in BCa tissues. Notably, the EZH2 expression pattern in tumor tissues was similar to that of SIRT7, suggesting that only the shRNA EZH2 lentivirus treatment could alter the EZH2 expression levels (Fig. 9D). On the other hand, CDDP treatment and the administration of the SIRT7 shRNA or EZH2 shRNA lentiviruses significantly upregulated RND3 in BCa tissues. Notably, under CDDP treatment, the combined application of the SIRT7 shRNA and EZH2 shRNA lentiviruses markedly enhanced the tumor RND3 protein levels compared to the administration of either shRNA lentivirus alone (Fig. 9D). These findings collectively suggest that shRNA lentiviruses targeting SIRT7 and EZH2 can significantly augment CDDP sensitivity and RND3 expression in BCa cells in vivo.

## Discussion

The reduced sensitivity of BCa cells to CDDP is a significant factor in the diminished efficacy of first-line platinum-based chemotherapy. Previous research has posited that SIRT7 could enhance the malignancy of BCa cells [8, 9], a phenomenon somewhat consistent with our findings. Specifically, we found that SIRT7 content in BCa tissues correlated with the sensitivity of BCa patients to platinum-based treatment. Furthermore, compared to normal cells, BCa cells exhibited a significantly higher expression of the SIRT7 protein. Additionally, SIRT7 knockdown influenced various cellular processes, resulting in reduced cell proliferation rate, decreased cellular activity and colony formation capacity, and increased apoptosis rates, with SIRT7 overexpression exerting opposite effects. Additionally, the IC50 value for CDDP correlated significantly positively with intracellular SIRT7 levels in BCa cell lines. Moreover, in vitro experiments demonstrated that inhibiting SIRT7 expression enhanced the cytotoxicity of CDDP in BCa cells. Moreover, the in vivo outcomes were consistent with the in vitro results, demonstrating that the BCa cell growth rate was significantly lower in the CDDP + SIRT7 shRNA lentivirus combination therapy group than in the CDDP alone group, suggests that targeting SIRT7 may enhance BCa cell CDDP sensitivity both in vitro *and* in vivo. According to previous research, the SIRT7 inhibitor 97491 can reduce SIRT7 deacetylase activity in a dose-dependent manner, stabilizing p53 and enhancing apoptotic effects, which may inhibit tumor progression [25]. Nonetheless, additional research is still required to further explore the potential of SIRT7 inhibitors, such as 97491, to improve BCa CDDP sensitivity.

It has also been established that SIRT7 mediates the deacetylation of histone H3K18, resulting in the transcriptional repression of tumor suppression-associated genes [12]. Our RNA sequencing analysis revealed that SIRT7 siRNA promoted the mRNA expression of 199 genes and inhibited the mRNA levels of 212 genes in UMUC3 cells, both with or without CDDP. Herein, the SIRT7-regulated DEGs were mainly involved in ECM-receptor interaction and focal adhesion pathways, among others. Notably, research has associated the ECM-receptor interaction with the progression of breast cancer [26] and lung cancer [27]. Furthermore, focal adhesion has been linked with BCa prognosis [28]. Based on these findings, it is plausible that SIRT7 may affect BCa cell growth and CDDP resistance by disrupting the aforementioned pathways, although this hypothesis requires further elucidation. Notably, RND3/RhoE, an atypical member of the Rho GTPase family, lacks GTP hydrolytic activity. Herein, SIRT7 knockdown significantly upregulated RND3 protein levels in BCa cells, with or without CDDP treatment. Furthermore, SIRT7 enrichment was observed in the RND3 gene promoter region, resulting in H3K18ac downregulation within this region. These findings suggests that SIRT7 can bind to the RND3 promoter, resulting in decreased H3K18ac levels, and ultimately in RND3 downregulation within the RND3 gene promoter region. The involvement of RND3 in cell cycle progression, cell survival, and apoptosis is well-documented [29]. For instance, compared to non-tumoral liver tissues, Hepatocellular Carcinoma (HCC) tissues exhibited a lower RND3 expression at both the mRNA and protein levels [30–32]. Furthermore, silencing RND3 inhibited the growth of HCC [33]. However, various malignancies, including Colorectal Cancer (CRC), Gastric Cancer (GC), and Non-Small Cell Lung Cancer (NSCLC) exhibited RND3 upregulaation [29]. Herein, RND3 inhibited the activity and clonogenic capacity of BCa cells, suggesting that its functions as a tumor suppressor in BCa. Additionally, RND3 knockdown attenuated the inhibitory effects of CDDP on the activity and clonogenic capacity of BCa cells, as well as CDDP-induced apoptosis, indicating that it increased BCa cell CDDP sensitivity. Furthermore, RND3 overexpression increased BCa cell CDDP sensitivity and RND3 knockdown inhibited the SIRT7 siRNA-mediated enhancement of BCa cell CDDP sensitivity. Moreover, RND3 overexpression abolished the SIRT7 overexpression-mediated inhibition of CDDP sensitivity in BCa cells. These findings suggests that SIRT7 suppressed RND3 expression via H3K18 deacetylation in the RND3 gene promoter region, promoting the proliferation of BCa cells and reducing their sensitivity to CDDP. Nonetheless, the involvement of RND3 in BCa cell growth and SIRT7 siRNA-induced increased CDDP sensitivity in vivo requires further experimental validation.

According to research, SIRT7 can regulate the metastatic abilities of cancer cells in various types of malignancies, including breast cancer [34], oral squamous cell carcinoma, [35] and prostate cancer [36]. Notably, the role of SIRT7 in BCa metastasis remains unclear. In our cohort for the IHC assay, before chemotherapy, the T stage and the ratios of LNM and vascular invasion were higher in BCa patients in the high SIRT7 content group than those in the low SIRT7 content group. This finding suggests the potential involvement of SIRT7 in BCa metastasis, although this needs to be investigated further. Since SIRT7 negatively regulated RND3 expression in BCa cells, we also detected RND3 expression in BCa tissues obtained from the above cohort using the IHC assay. According to the results, RND3 correlated significantly with vascular invasion. In a previous study, KIAA1429-mediated metastasis was found to be dependent on RND3 down-regulation in HCC [37]. Furthermore, the Epstein-Barr Virus (EBV) was found to inhibit RND3 expression, promoting nasopharyngeal carcinoma metastasis [38]. It is also noteworthy that RND3 can regulate the RhoA /ROCK pathway [39, 40]. Moreover, ROCK-myosin was found to drive the fast rounded-amoeboid migration in cancer cells during metastatic dissemination [41]. Based on these research insights, we speculated that RND3 can affect the migratory ability of BCa cells, particularly exerting an inhibitory effect, which might contribute to BCa metastasis. Although RND3 could regulate the migratory ability of BCa cells, additional experimental research will be required to establish whether this effect depends on the RHOA/ROCK pathway. We also found that RND3 expression correlated significantly negatively with SIRT7 content in BCa tissues, implying the potential involvement of RND3 in SIRT7-mediated BCa metastasis.

In previous research, EZH2 catalyzed the mono-, di-, and tri-methylation of histone H3K27, silencing various genes [42], including the tumor suppressor genes, thus facilitating the progression of cancer cells in various malignancies, including the breast [43] and gastric [44] cancers, and conferring resistance to CDDP chemotherapy [45, 46]. Recent research has also indicated that EZH2 is intricately associated with BCa recurrence [47] and metastasis [48], primarily due to its regulatory influence on critical biological processes, including BCa cell apoptosis [49], proliferation, and migration [50]. Through epigenetic modification, EZH2 can silence the expression of genes such as CDH1 [51] and ARHGDIB [48], promoting BCa progression. Furthermore, EZH2 can downregulate the expression levels of genes such as CBX7 [17] and miR-194-5p [18], diminishing BCa cell CDDP sensitivity. Herein, EZH2 upregulation correlated with BCa cell CDDP sensitivity, whereas EZH2 upregulation enhanced CDDP sensitivity in these cells. Additionally, EZH2 could be recruited to the RND3 gene promoter region, increasing H3K27me^3^ levels and subsequently suppressing RND3 expression. Moreover, RND3 expression inhibition could obstruct the increased BCa cell CDDP sensitivity achieved via EZH2 knockdown. We also found that EZH2 overexpression promoted BCa cell growth and CDDP resistance, a phenomenon that RND3 overexpression reversed. These findings suggest that EZH2 can modulates the H3K27me^3^ levels in the RND3 gene promoter region, resulting in this gene’s transcriptional repression and ultimately diminishing BCa cell CDDP sensitivity. Notably, the elucidation of this pathway illuminates the molecular mechanism by which EZH2 influences BCa cell CDDP sensitivity. Simultaneously, EZH2 and SIRT7 collaboratively suppressed the expression of the RND3 gene by modulating the levels of H3K27me^3^ and H3K18ac, respectively, indicating that a convergent regulatory pathway influenced BCa cell CDDP sensitivity.

Interestingly, we also found a significant positive correlation between SIRT7 and EZH2 levels in BCa cell lines. In our analysis, SIRT7 and EZH2 appeared to influence each other’s recruitment to the RND3 gene promoter region, resulting in H3K18ac suppression and H3K27me^3^ promotion. Specifically, SIRT7 bound to EZH2, a phenomenon that enhanced their respective roles in epigenetic modifications, thus contributing to RND3 expression inhibition and BCa cell CDDP resistance (Fig. 7M). Multiple studies have shown that epigenetic modifications on chromosomes can influence the binding affinity of DNA-binding proteins and crucially regulate gene expression. For instance, the zinc finger domain protein SALL4A exhibited a preference for binding to DNA containing 5-hydroxymethylcytosine (5hmC) modifications, facilitating further oxidation of 5hmC and contributing to gene expression regulation [52]. Additionally, TET2, an enzyme responsible for DNA hydroxylation, interacted with histone deacetylase HDAC2, promoting histone deacetylation and subsequently mediating IL-6 expression inhibition [53]. Nonetheless, whether the potential reciprocal promotion of SIRT7 and EZH2 recruitment to the RND3 promoter region depends on binding interactions remains to be elucidated. Furthermore, it is yet to be experimentally confirmed whether the SIRT7-mediated H3K18ac downregulation or the EZH2-induced H3K27me^3^ upregulation can facilitate the recruitment of EZH2 and SIRT7 proteins to the RND3 promoter, respectively.

In recent reports, SIRT7 exerted post-translational regulatory functions on non-histone proteins, including deacetylation [21–23] and desuccinylation [24]. Herein, SIRT7 bound to the EZH2 protein and its knockdown significantly upregulated the succinylation, rather than acetylation, of lysine residues on the EZH2 protein. Succinyl-CoA, which mediates EZH2 succinylation, was found to reduce the abundance of the EZH2 protein and H3K27me^3^ levels in the promoter region of the RND3 gene. This finding suggests that SIRT7 promoted EZH2 desuccinylation, facilitating the recruitment of the EZH2 protein to the RND3 promoter, thus increasing the levels of H3K27me^3^ in this region. Additionally, fibrillin 1 was found to predominantly exist in a K672-succinylated form in GC, impeding its interaction with MMP2, resulting in the inhibition of its degradation and accumulation [54]. Furthermore, although succinylation at the K311 site of the glutaminase protein can enhance its oligomerization and activity, its expression levels are only minimally impacted [55]. Herein, neither SIRT7 knockdown nor succinyl-CoA stimulation significantly altered the expression levels of the EZH2 protein in BCa cells. This finding suggests that the SIRT7-mediated reduction in EZH2 protein succinylation may alter EZH2 function, facilitating its recruitment to the RND3 gene promoter region. Nonetheless, additional research will be required to establish whether the structural integrity and nuclear localization capability of the EZH2 protein were affected.

Besides RND3, we also explored other possible mechanisms through which SIRT7 and EZH2 synergistically regulate the growth and CDDP sensitivity of BCa cells. Herein, Protein-Protein Interaction (PPI) analysis showed that SIRT7 regulated nine DEGs, including H2BC12, POLR2A, POLR2G, POLR2K, POLR1E, MEF2D, TBL1X, KMT2D, and CREBBP. Notably, all these DEGs correlated with SIRT7 and EZH2, directly or indirectly. According to research, POLR2A is crucially involved in the pathogenesis of breast cancer [56] and glioblastoma [57]. Furthermore, MEF2D has been implicated in the growth and metastasis of liver cancer [58], gastric cancer [59], and pancreatic cancer [60]. Moreover, in liver cancer, the effects of SIRT7 on the efficacy of the checkpoint inhibitor were found to be dependent on MEF2D [23]. Based on these insights, we speculated that except for RND3, SIRT7 and EZH2 collaborate to regulate POLR2A and MEF2D, either directly or indirectly, leading to BCa progression and CDDP resistance.

In recent cancer therapy, there has been a progressive transition from mono-therapy to combination therapy to effectively combat heterogeneous cancer cells and the complex Tumor Microenvironment (TME) [61]. For instance, to optimize cancer treatment and achieve improved clinical outcomes, active compounds derived from Traditional Chinese Medicine (TCM) are presently being integrated with various cancer treatment strategies, including chemotherapy, gene therapy, radiotherapy, phototherapy, and immunotherapy, [62]. Moreover, the combination of immune checkpoint inhibitors with interleukins and interleukin-targeting agents yield promising preliminary results [63]. Herein, silencing SIRT7 and EZH2 expression via siRNA significantly inhibited BCa cell proliferation (as evidenced by the reduced cell viability and clonogenic capacity) and increased apoptosis rates. Furthermore, this intervention enhanced BCa cell CDDP sensitivity in vitro. Notably, the combined silencing of SIRT7 and EZH2 exerted a more pronounced regulatory effect compared to the silencing of either SIRT7 or EZH2 alone. In a BCa animal model established via subcutaneous injection of BCa cells into Balb/c nude mice, the combined treatment targeting SIRT7 and EZH2 using shRNA lentivirus significantly enhanced the inhibitory effect of CDDP on BCa tissue growth. Compared to the application of a single shRNA lentivirus, this combined approach exerted a more potent regulatory effect. These findings suggest that the concurrent inhibition of SIRT7 and EZH2 expression markedly increases BCa cell CDDP sensitivity. Nonetheless, it is noteworthy that both siRNA and shRNA lentivirus therapies are currently at the research stages and are yet to be translated into clinical practice. Furthermore although several EZH2 inhibitors, such as Ezharmia (valemetostat), Tazverik, and Amisole, have already been approved for clinical use, it remains unclear whether they can enhance BCA cell CDDP sensitivity by targeting SIRT7 and EZH2.

Herein, the cytotoxicity of CDDP on BCa cells was contingent upon RND3 upregulation. Although CDDP did not alter the SIRT7 and EZH2 protein levels in BCa cells, it reduced the enrichment of these proteins at the RND3 promoter region. This effect was accompanied by H3K18ac upregulation and H3K18me^3^ downregulation at the same promoter region. These findings suggest that the regulatory effect of CDDP on RND3 expression correlated with the reduced recruitment of SIRT7 and EZH2 to the RND3 gene promoter region. At the same time, CDDP treatment enhanced EZH2 protein succinylation in BCa cells. In previous research, CDDP-induced Dicer in HEK293T cells interacted with SIRT7, sequestering SIRT7 in the cytoplasm and preventing SIRT7-induced H3K18ac downregulation [13]. Furthermore, Dicer content was reduced in CDDP-resistant ovarian cancer cells and its inhibition diminished the sensitivity of ovarian cancer cells to CDDP [64]. Based on these insights, we speculated that CDDP treatment may promote the expression of Dicer, which can bind to SIRT7, facilitating the relocation of SIRT7 to the cytoplasm and the inhibition of SIRT7-mediated H3K18 de-acetylation at the RND3 promoter. Furthermore, it remains unknown whether the CDDP-induced Dicer upregulation can block the binding of SIRT7 and EZH2 proteins, thus inhibiting EZH2 protein de-succinylation and decreasing EZH2 enrichment in the RND3 gene promoter region.

In summary, this study revealed the regulatory role and related mechanisms of SIRT7 and EZH2 in jointly inhibiting RND3 expression via epigenetic modification, thus mediating decreased BCa cell CDDP sensitivity. Despite its valuable insights, this study had certain limitations. First, further research will be required to elucidate the role of EZH2 in BCa progression and its impact on CDDP sensitivity in clinical samples. Nonetheless, we found that SIRT7 expression in BCa tissues correlated significantly with both BCa progression and sensitivity to CDDP. Second, we did not generate SIRT7 and EZH2 overexpression plasmids to verify the inhibitory effect of SIRT7 on BCa cell CDDP sensitivity. Instead, we focused on the effects of targeting SIRT7 and EZH2 in the high-expression BCa cell line by constructing specific siRNAs for each gene to assess their impact on the cell growth and BCa cell CDDP sensitivity. Simultaneously, the primary objective of this study was to establish whether the combined inhibition of SIRT7 and EZH2 could enhance BCa cell CDDP sensitivity. Additionally, the potential of SIRT7 and EZH2 inhibitors to improve BCa cell CDDP sensitivity was not investigated, warranting additional research in the future.

## Conclusion

Overall, SIRT7 and EZH2 modulated the levels of H3K18ac and H3K27me^3^ within the RND3 gene promoter region, respectively. Furthermore, they collaboratively suppressed RND3 gene expression, thus contributing to CDDP resistance in BCa cells. Moreover, SIRT7 and EZH2 synergistically enhanced each other’s regulatory functions, a phenomenon attributable to the SIRT7-mediated de-succinylation of the EZH2 protein. Therefore, targeting SIRT7 and EZH2 may represent a viable therapeutic avenue for enhancing BCa cell CDDP sensitivity.

## Supplementary information

Supplementary Figure Legends

Supplementary Figure 1

Supplementary Figure 2

Supplementary Figure 3

Supplementary Figure 4

WB Original Data

## Data Availability

The datasets used and analyzed during the current study are available from the corresponding author upon reasonable request.
