# Supplementary material for: Mechanistic insights into SIRT7 and EZH2 regulation of cisplatin resistance in bladder cancer cells
Source: Cell Death Dis. 2024 Dec 24;15(12):931. doi: 10.1038/s41419-024-07321-1 (PMC11668892; doi:10.1038/s41419-024-07321-1)
Supplement: Supplementary file 1 — Supplementary Figure Legends [file 41419_2024_7321_MOESM1_ESM.docx]

**Supplementary Figure Legends**

**Supplementary Figure 1. Kaplan-Meier curve comparing the DFS between high and low SIRT7 expression groups of bladder cancer in patients from the TCGA cohort.** A cohort of 117 bladder cancer patients who had not received platinum-based chemotherapy, with their SIRT7 expression and survival data were analyzed. Statistical analysis and plots were performed on the R software. The Kaplan-Meier curve function was employed to compare the DFS between patients with high SIRT7 expression and those with low SIRT7 level, with survival curves generated accordingly. The survival rates were compared using the log-rank test. Statistical significance was defined as a p< 0.05.

**Supplementary Figure 2. GO analysis and KEGG enrichment analysis were performed to predict the genes regulated by SIRT7 siRNA.** GO analysis (A) and KEGG (C) enrichment analyses were conducted on the 199 genes which were up-regulated in UMUC3 cells following treatment with SIRT7 siRNA, either with or without CDDP. GO analysis (B) and KEGG (D) enrichment analyses were conducted on the 212 genes that were down-regulated in UMUC3 cells following treatment with SIRT7 siRNA, either with or without CDDP.

**Supplementary Figure 3. The effects of RND3, SIRT7, and EZH2 overexpression plasmids on the growth and CDDP sensitivity of TCCSUP cells.** (A-C) TCCSUP cells were transfected with negative control, RND3, SIRT7, and EZH2 overexpression plasmids and treated with or without CDDP. (A) The protein levels of RND3, SIRT7, and EZH2 was determined by Western Blot. (B) CCK-8 assays were performed to measure cell viability. (C) Flow cytometry was conducted to evaluate apoptosis of TCCSUP cells. Quantitative data are presented as mean ± SD, n = 3. One-way ANOVA was used to assess group differences, with ns indicating p>0.05, and ** indicating p<0.01.

**Supplementary Figure 4. A PPI network was constructed for EZH2 and SIRT7, its potential target genes.** Firstly, 199 up-regulated and 212 down-regulated genes regulated by SIRT7 siRNA in UMUC3 cells, either with or without CDDP, were selected. The genes that interacted with SIRT7 and EZH2 directly or indirectly were determined on the STRING database. Finally, the PPI network based on SIRT7 and its target genes that were finally selected, and EZH2 was drawn using the Cytoscape.
