# Supplementary figures and images for: Mechanistic insights into SIRT7 and EZH2 regulation of cisplatin resistance in bladder cancer cells
Source: Cell Death Dis. 2024 Dec 24;15(12):931. doi: 10.1038/s41419-024-07321-1 (PMC11668892; doi:10.1038/s41419-024-07321-1)

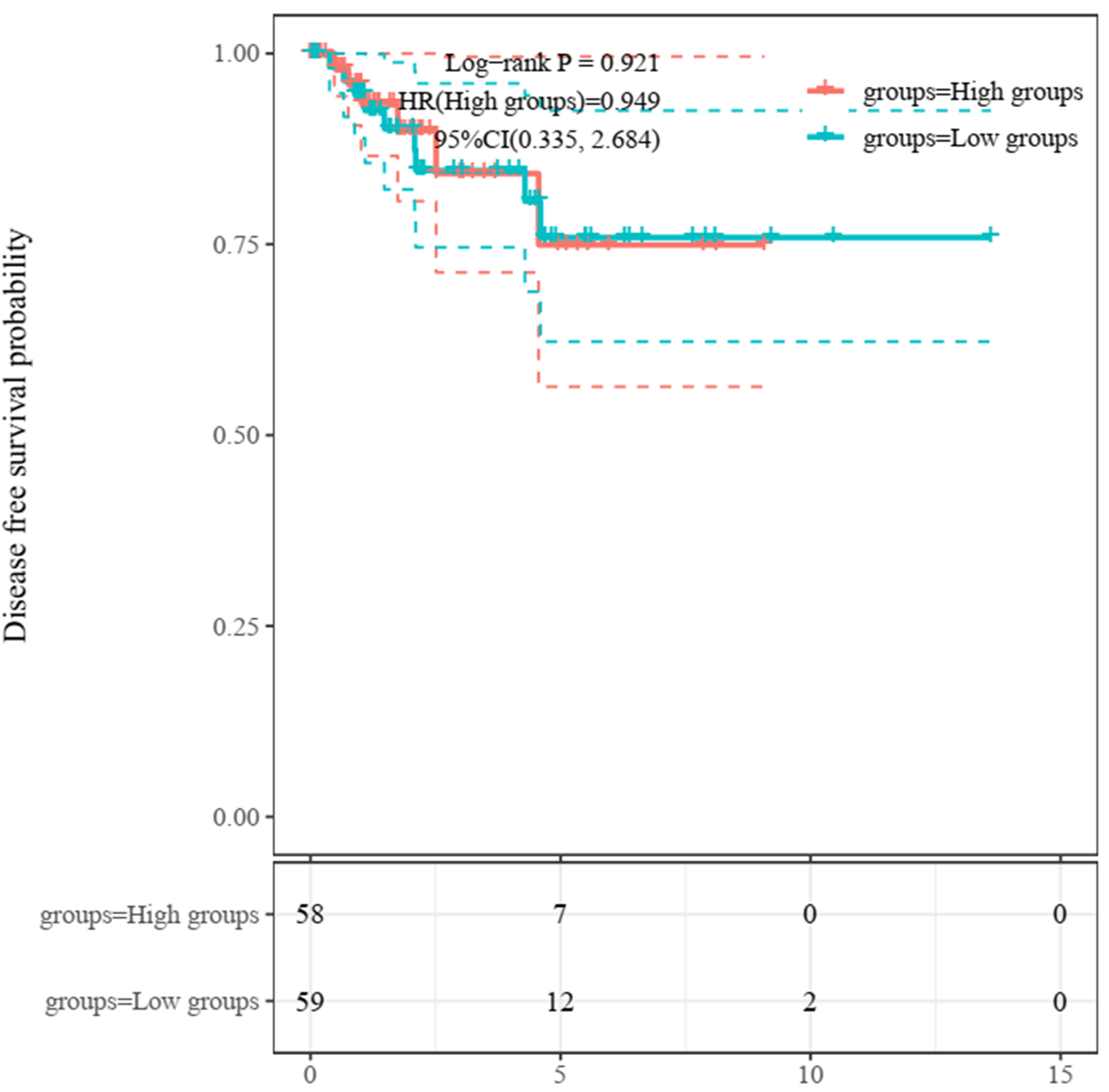

Supplement: Supplementary file 2 — Supplementary Figure 1 [file 41419_2024_7321_MOESM2_ESM.tif]

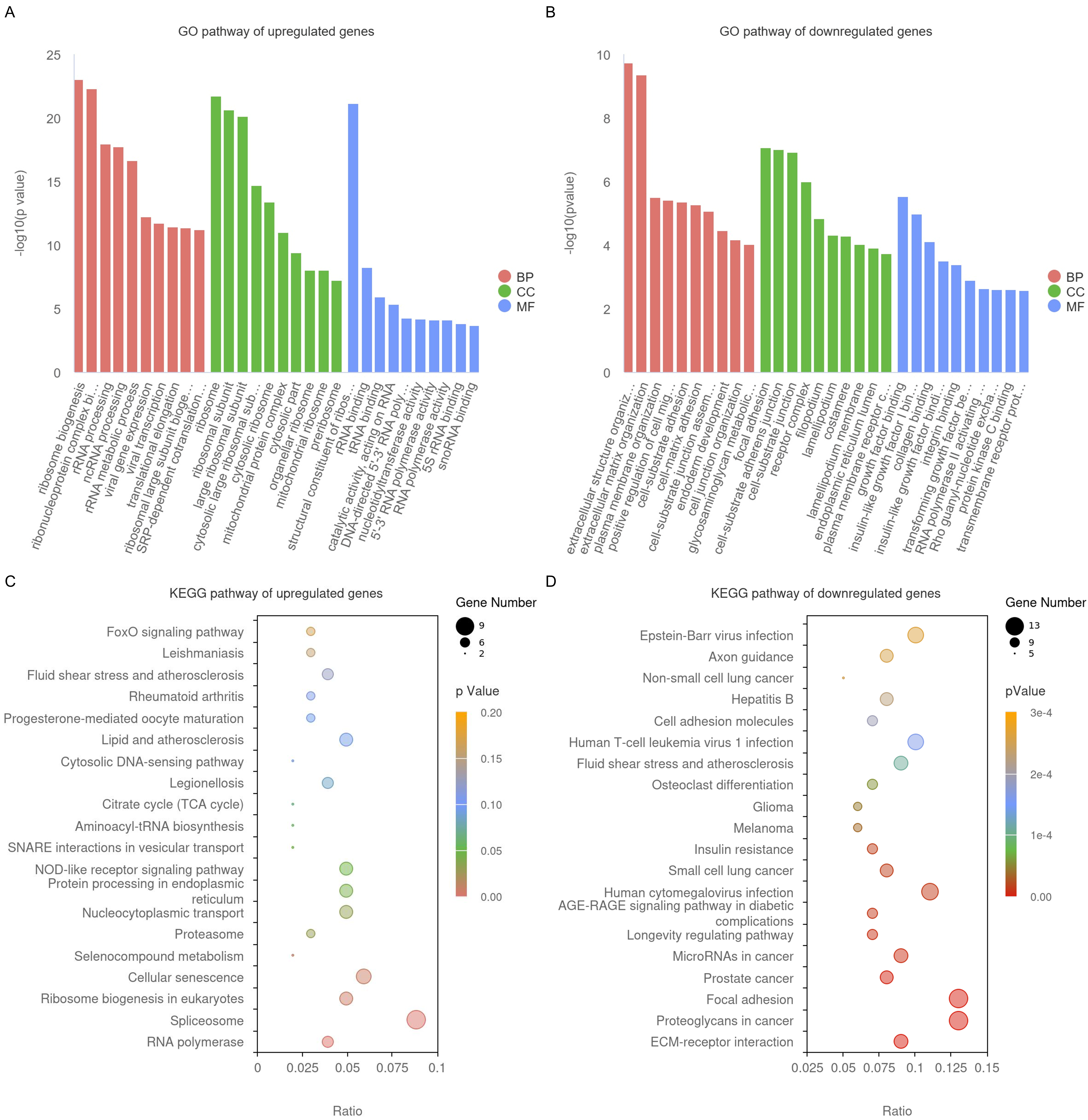

Supplement: Supplementary file 3 — Supplementary Figure 2 [file 41419_2024_7321_MOESM3_ESM.tif]

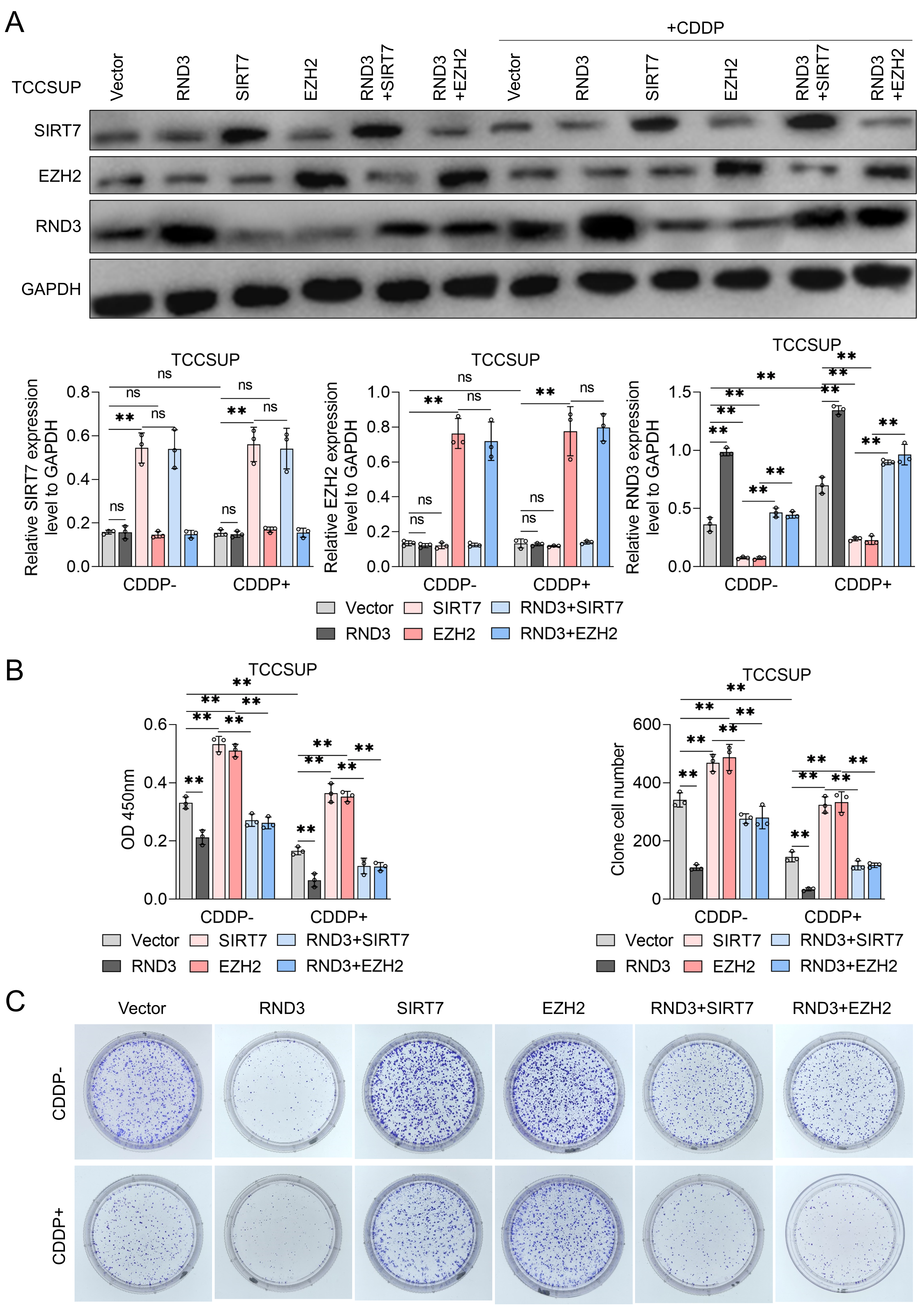

Supplement: Supplementary file 4 — Supplementary Figure 3 [file 41419_2024_7321_MOESM4_ESM.tif]

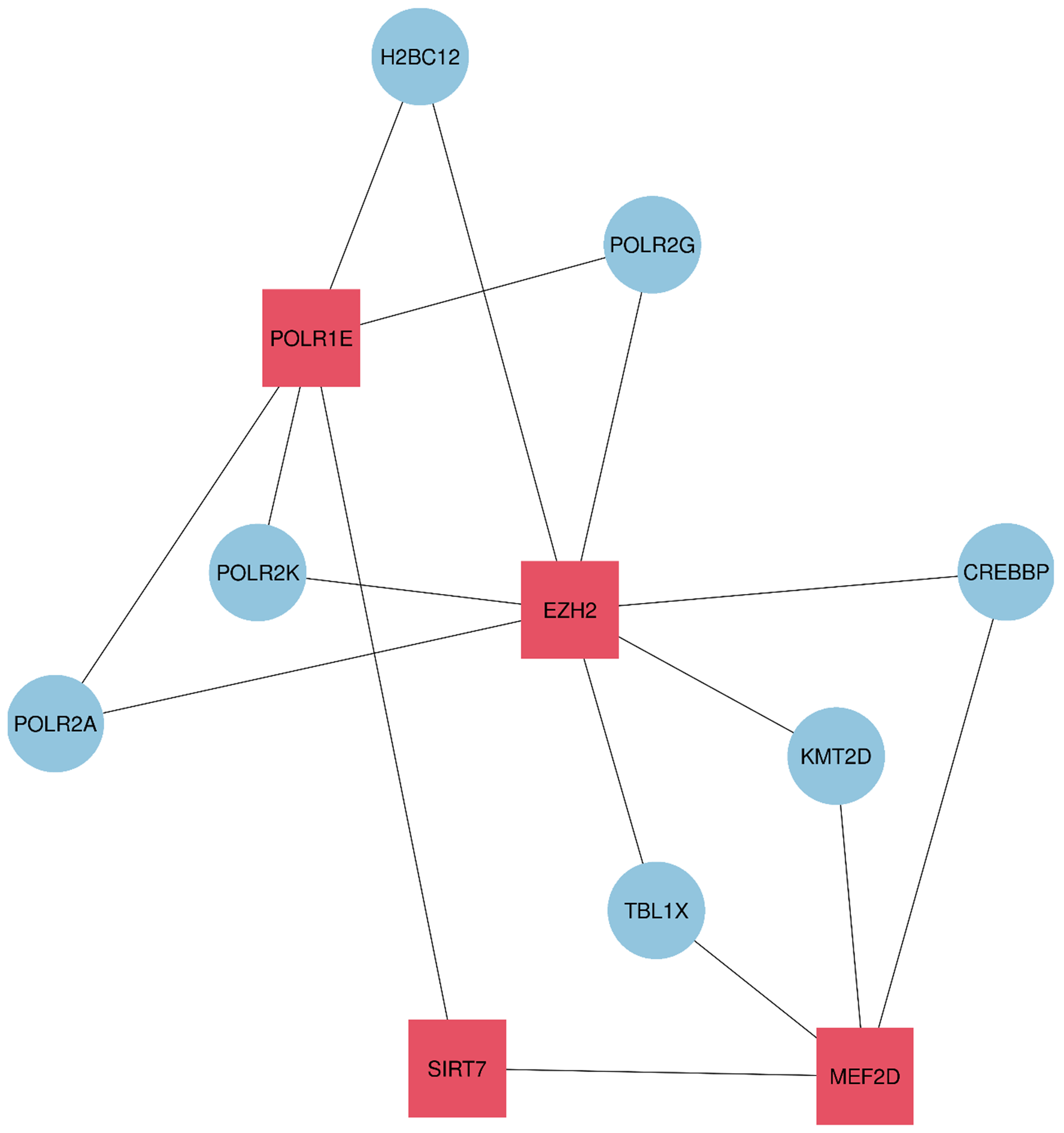

Supplement: Supplementary file 5 — Supplementary Figure 4 [file 41419_2024_7321_MOESM5_ESM.tif]
