# Supplementary material for: Mechanistic insights into SIRT7 and EZH2 regulation of cisplatin resistance in bladder cancer cells
Source: Cell Death Dis. 2024 Dec 24;15(12):931. doi: 10.1038/s41419-024-07321-1 (PMC11668892; doi:10.1038/s41419-024-07321-1)
Supplement: Supplementary file 6 — WB Original Data [file 41419_2024_7321_MOESM6_ESM.pdf]

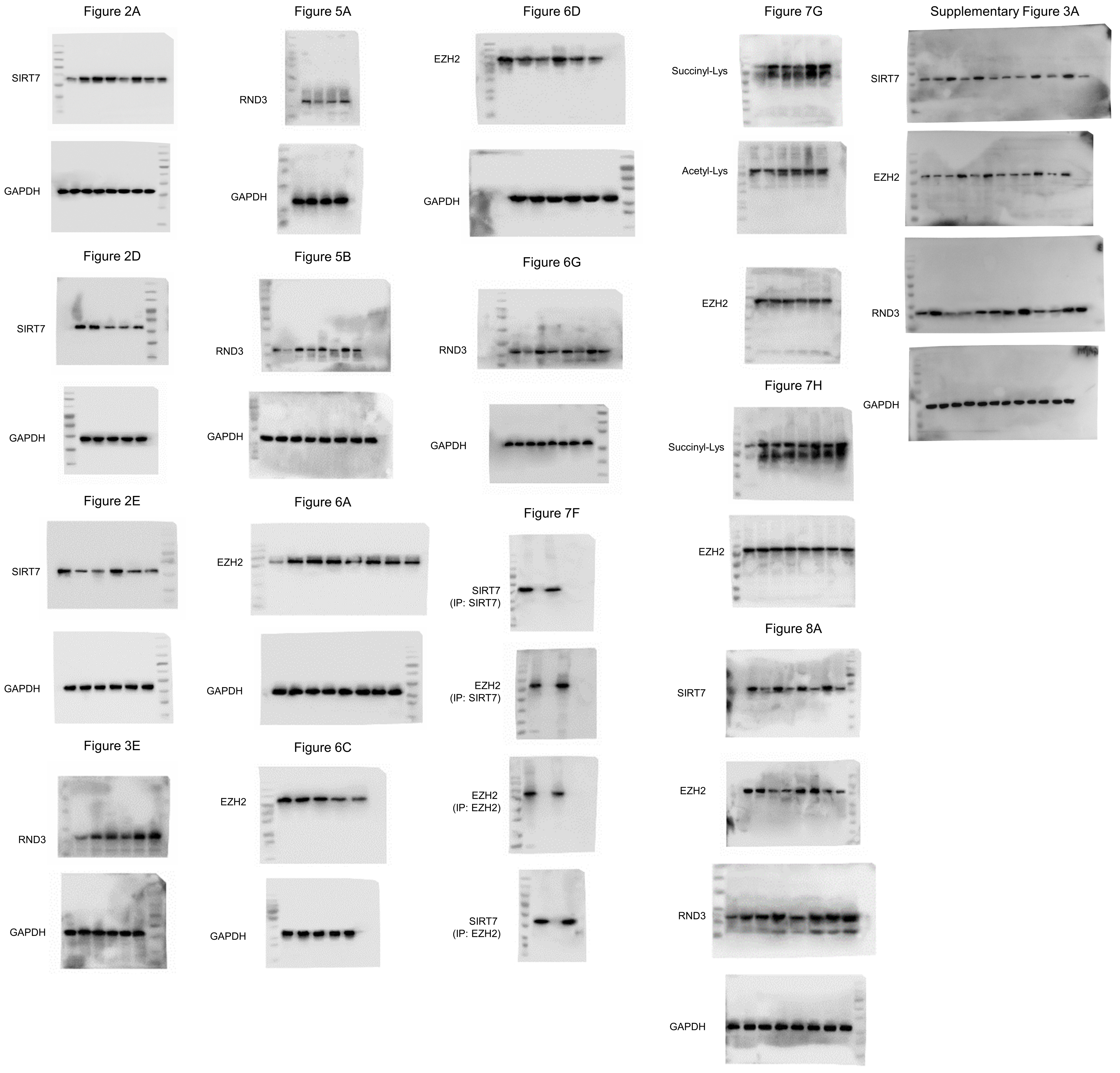
Figure 2A
SIRT7
GAPDH
Figure 2D
SIRT7
GAPDH
Figure 2E
SIRT7
GAPDH
Figure 3E
RND3
GAPDH
Figure 5A
RND3
GAPDH
Figure 5B
RND3
GAPDH
Figure 6A
EZH2
GAPDH
Figure 6C
EZH2
GAPDH
Figure 6D
EZH2
GAPDH
Figure 6G
RND3
GAPDH
Figure 7F
SIRT7 (IP: SIRT7)
EZH2 (IP: SIRT7)
EZH2 (IP: EZH2)
SIRT7 (IP: EZH2)
Figure 7G
Succinyl-Lys
Acetyl-Lys
EZH2
Figure 7H
Succinyl-Lys
EZH2
Figure 8A
SIRT7
EZH2
RND3
GAPDH
Supplementary Figure 3A
SIRT7
EZH2
RND3
GAPDH
